# A Deep‐Blue‐Emitting Heteroatom‐Doped MR‐TADF Nonacene for High‐Performance Organic Light‐Emitting Diodes**

**DOI:** 10.1002/anie.202215522

**Published:** 2023-01-16

**Authors:** Subeesh Madayanad Suresh, Le Zhang, David Hall, Changfeng Si, Gaetano Ricci, Tomas Matulaitis, Alexandra M. Z. Slawin, Stuart Warriner, Yoann Olivier, Ifor D. W. Samuel, Eli Zysman‐Colman

**Affiliations:** ^1^ Organic Semiconductor Centre, EaStCHEM School of Chemistry University of St Andrews St Andrews KY16 9ST UK; ^2^ Organic Semiconductor Centre SUPA School of Physics and Astronomy University of St Andrews St Andrews KY16 9SS UK; ^3^ School of Chemistry University of Leeds Woodhouse Lane Leeds LS2 9JT UK; ^4^ Laboratory for Computational Modeling of Functional Materials & Solid State Physics Laboratory Namur Institute of Structured Matter University of Namur Rue de Bruxelles, 61 5000 Namur Belgium

**Keywords:** Deep Blue Emission, Multi-Resonance, Nanographene, Organic Light-Emitting Diode, Thermally Activated Delayed Fluorescence

## Abstract

We present a p‐ and n‐doped nonacene compound, NOBNacene, that represents a rare example of a linearly extended ladder‐type multiresonant thermally activated delayed fluorescence (MR‐TADF) emitter. This compound shows efficient narrow deep blue emission, with a *λ*_PL_ of 410 nm, full width at half maximum, FWHM, of 38 nm, photoluminescence quantum yield, Φ_PL_ of 71 %, and a delayed lifetime, *τ*_d_ of 1.18 ms in 1.5 wt % TSPO1 thin film. The organic light‐emitting diode (OLED) using this compound as the emitter shows a comparable electroluminescence spectrum peaked at 409 nm (FWHM=37 nm) and a maximum external quantum efficiency (EQE_max_) of 8.5 % at Commission Internationale de l’Éclairage (CIE) coordinates of (0.173, 0.055). The EQE_max_ values were increased to 11.2 % at 3 wt % doping of the emitter within the emissive layer of the device. At this concentration, the electroluminescence spectrum broadened slightly, leading to CIE coordinates of (0.176, 0.068).

## Graphical abstract

A deep blue boron‐, nitrogen‐ and oxygen‐doped nonacene emitter that shows multi‐resonant thermally activated delayed fluorescence (MR‐TADF) is described. The emitter shows bright, deep‐blue narrowband emission in an organic light emitting diode (OLED) device.

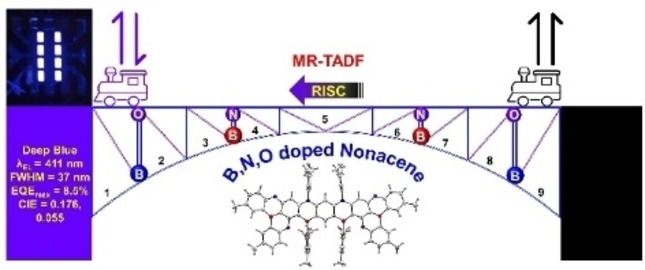

## Introduction

The performance of organic light‐emitting diodes (OLEDs) has advanced significantly since the first OLED developed by Tang and Van Slyke more than 30 years ago.^[1]^ OLED technology is now widely adopted in a range of consumer electronics such as mobile phones, smart watches, and televisions. Singlet and triplet excitons are formed in an electroluminescent (EL) device in the ratio 1 : 3 due to the Fermionic nature of holes and electrons.^[2]^ Commercial displays use two classes of emitters for the primary colors.^[1b]^ Phosphorescent emitters are used for green and red, whereas triplet‐triplet annihilation (TTA) using purely organic emitters are used for the blue emitter as there is at present not a sufficiently stable blue phosphorescent OLED.^[3]^ OLEDs with TTA emitters, however, can only achieve a maximum 62.5 % internal quantum efficiency (IQE), thus, there is still room for improvement of the overall efficiency of the device. Not surprisingly, there is a huge effort undertaken by both academia and industry to develop stable and high efficiency blue emitters that can harvest 100 % IQE in the device.^[3]^ Furthermore, deep blue OLED light sources can find application beyond displays,^[4]^ such as for sterilization,^[5]^ and dental^[6]^ and dermatological treatments.^[7]^ Purely organic thermally activated delayed fluorescent (TADF) emitters provide a tantalizing solution as TADF OLEDs can achieve up to 100 % IQE.^[8]^ However, donor‐acceptor (D‐A) TADF compounds show very broad emission due to the long‐range charge‐transfer (LRCT) character of the excited state and the broad range of accessible geometries in the excited state as the D and A units are connected through single bonds.^[9]^ This results in OLEDs that show poor color purity.^[10]^ A solution to this apparent weakness of D‐A TADF emitters was advanced by Hatakeyama et al. who demonstrated how p‐ and n‐doped nanographenes, termed multi‐resonant TADF (MR‐TADF) emitters, could also exhibit TADF but with much narrower emission profiles.^[11]^ The narrow emission spectra were rationalized in terms of the rigid structure of these compounds together with the short‐range charge transfer (SRCT) nature of the emissive excited state.^[9]^

**Figure 1:**
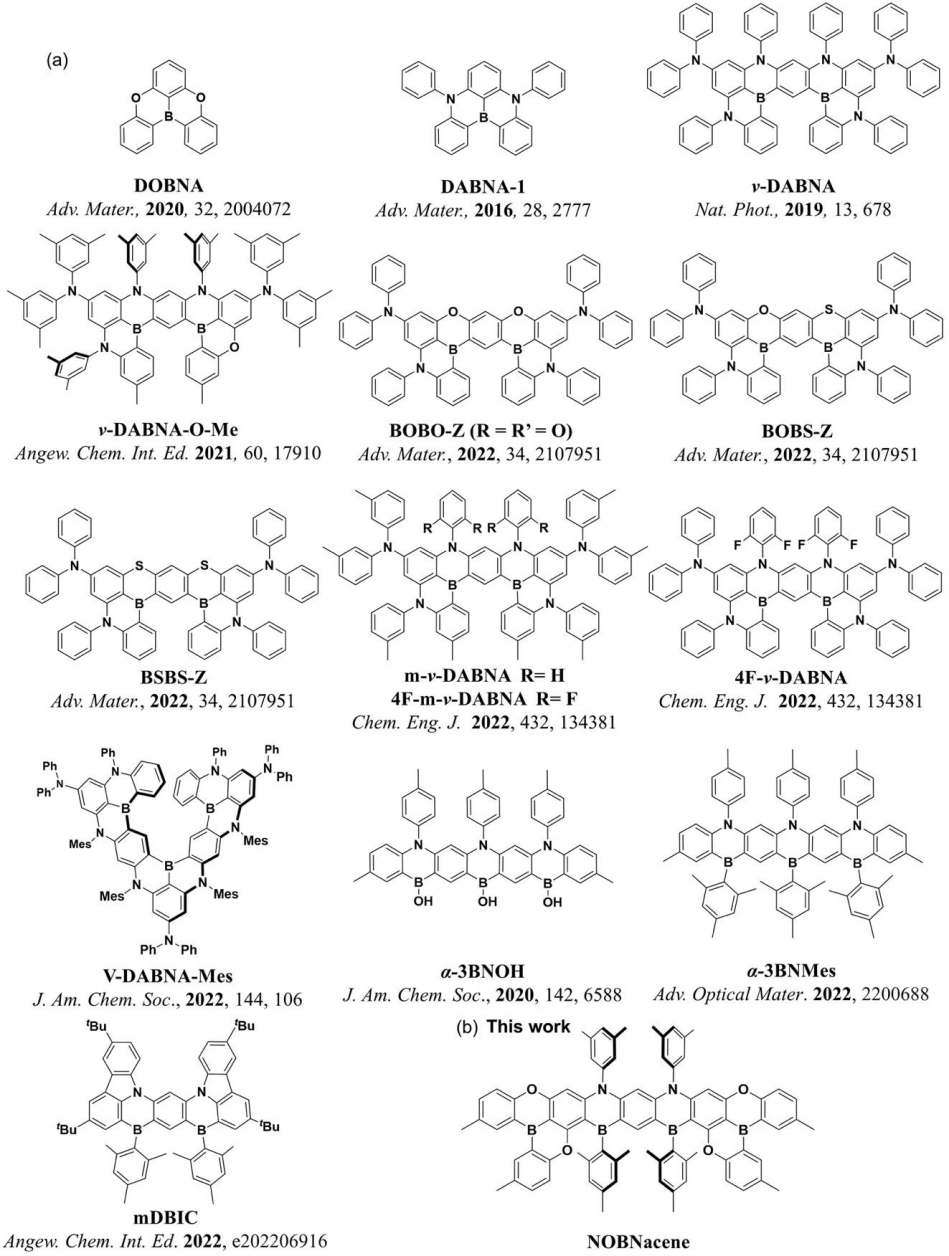
Chemical structures of a) DOBNA, DABNA‐1 and reported π‐extended blue MR‐TADF emitters. b) NOBNacene.

The potential of MR‐TADF compounds to act as pure blue OLED emitters was first exemplified by the DABNA series (Figure 1).^[11]^ DABNA‐1 presents a photoluminescence maximum, *λ*_PL_, of 460 nm, a full width at half maximum, FWHM, of 30 nm, a high photoluminescence quantum yield, Φ_PL_, of 88 %, and a moderate singlet‐triplet energy gap, Δ*E*_ST_, of 180 meV in 1 wt % mCBP host. The corresponding OLED showed pure blue emission at *λ*_EL,_ of 460 nm, with a full width at half maximum, FWHM, of 30 nm, corresponding to a Commission Internationale de l’Éclairage (CIE) y‐coordinate of 0.09, and a moderately high maximum external quantum efficiency, EQE_max,_ of 13.5 %. The same group reported a π‐extended version of DABNA‐1, *v*‐DABNA, which still represents the pinnacle of blue MR‐TADF emitter design.^[12]^ This emitter exhibited efficient narrowband blue emission (*λ*_PL_=467 nm, FWHM=18 nm, Φ_PL_=90 %) and a very small Δ*E*_ST_ of 17 meV in 1 wt % DOBNA‐OAr host. The OLED showed an impressive performance with an EQE_max_ as high as 34.4 % at *λ*_EL_ of 469 nm, a FWHM of 18 nm and CIE coordinates of (0.12, 0.11). Indeed, compared to the OLED with DABNA‐1, there is a remarkable improvement in the device performance with *v*‐DABNA. The same group modified the structure of *v*‐DABNA by replacing one of the nitrogen atoms with a less electron‐donating oxygen atom as in *v*‐DABNA‐OMe.^[13]^ This emitter maintained efficient and narrowband blue emission (*λ*_PL_=464 nm, FWHM=24 nm in 1 wt % DABNA‐OAr). The OLED with this derivative showed a slightly blue‐shifted EL of 465 nm (FWHM=23 nm, EQE_max_=29.5 %). Kwon et al. modified the *v*‐DABNA core to incorporate methyl and electron‐withdrawing fluorine substituents.^[14]^ Among their three emitters, 4F‐*m*‐*v*‐DABNA (*λ*_PL_=455 nm, FWHM=14 nm in PhMe) exhibited the most hypsochromic emission compared to the parent *v*‐DABNA (*λ*_PL_=467 nm, 1 wt % doped in DABNA‐OAr).^[15]^ OLEDs with 4F‐*m*‐*v*‐DABNA showed EQE_max_ of 33.7 % and an impressive CIE coordinate of (0.13, 0.06).^[14]^ Yasuda et al. investigated doping mixed donor (oxygen and sulfur) atoms within the *v*‐DABNA core to tune the emission into the deep blue region.^[16]^ The new emitters all presented blue‐shifted emission compared to the parent *v*‐DABNA (*λ*_PL_=474 nm, 3 wt % doped in mCBP). Their bluest emitter, BOBO‐Z, emits at 445 nm, with the same FWHM of 18 nm. Both Δ*E*_ST_ and the delayed lifetime (*τ*_d_) increased to 0.102 eV, and 7.7 μs, respectively. The device with this emitter showed deep blue EL (*λ*_EL_=445 nm, FWHM=18 nm, EQE_max_=13.6 %, CIE_y_=0.04). Recently Hatakeyama et al. reported a π‐extended B,N‐doped helicene, *v*‐DABNA‐Mes that showed efficient and narrowband PL at *λ*_PL_=484 nm, FWHM=16 nm (Δ*E*_ST_=8.5 meV, *τ*_D_=2.4 μs, Φ_PL_=80 %, 1 wt % doped in PMMA).^[17]^ The OLEDs emitted at *λ*_EL_=480 nm (FWHM=27 nm; CIE_y_=0.21) and showed an EQE_max_=22.9 %. However, the emission was red‐shifted to the sky‐blue region due to its extended π‐conjugation. We recently reported a B,N‐doped heptacene, α‐3BNMes, that shows narrowband blue emission (*λ*_PL_=442 nm, FWHM=30 nm) in THF.^[18]^ The hyperfluorescent (HF)^[19]^ device with *α*‐3BNMes as a terminal emitter sustained blue EL (*λ*_EL_=443 nm, CIE_y_=0.1) with an EQE_max_ of 15 %. Recently, Duan et al. reported a B,N‐doped pentacene emitter, mDBIC, that emits in the deep blue (*λ*_PL_=426 nm, FWHM=26 nm) region in 2 wt % doped mCP film and was used as the terminal emitter in a HF device that showed excellent performance at the deep blue color point (*λ*_EL_=431 nm, FWHM=42 nm, EQE_max_=13.5 %, CIE_y_=0.05).^[20]^ Pushing the emission further into the deep blue in MR‐TADF compounds remains exceedingly difficult. The B,N‐doped heptacene system α‐3BNOH (*λ*_PL_=390 nm, Δ*E*_ST_=0.30 eV, *τ*_d_=0.45 μs, THF)^[21]^ that we previously reported represents a rare example of a MR‐TADF purple emitter as does the B,O‐doped triangulene emitter, DOBNA (*λ*_PL_=398 nm, Δ*E*_ST_=0.18 eV, *τ*_d_=66 μs, 1 wt % in PMMA).^[22]^

Building on our recent efforts to design linearly extended MR‐TADF acene emitters, here we report a boron‐, nitrogen‐, and oxygen‐doped polycyclic aromatic hydrocarbon that contains nine annulated six‐membered rings, NOBNacene, (Figure 1b). Despite the apparent large conjugation length, maintaining a regioregularity of the heteroatoms, and using oxygen donors rather than more electron‐donating nitrogen atoms results in a deep blue emission in this system. This compound represents arguably the first example of an MR‐TADF emitter possessing both electronically distinct donor (N and O) and acceptor groups (two distinct B atoms).[^12^, ^23^] In 1.5 wt % TSPO1 doped films, NOBNacene shows narrowband emission with a *λ*_PL_ of 410 nm, a FWHM of 38 nm, a high Φ_PL_ of 71 %, and a thermally activated delayed fluorescence with a *τ*_d_ of 1.18 ms. The Δ*E*_ST_ was measured to be 0.30 eV. The corresponding deep blue OLED shows outstanding performance with a maximum external quantum efficiency (EQE_max_) of 8.5 % at a peak electroluminescence (*λ*_EL_) of 409 nm (FWHM of 37 nm), with corresponding to Commission Internationale de l’Éclairage coordinates of (0.173, 0.055), very close to the BT.2020 requirement for the blue pixel of (0.131, 0.046).^[24]^ NOBNacene combines the benefits of high efficiency, narrow near‐UV emission, and high color purity, representing a promising emitter design approach to high‐performance deep blue OLEDs.

## Results and Discussion

**Figure 2:**
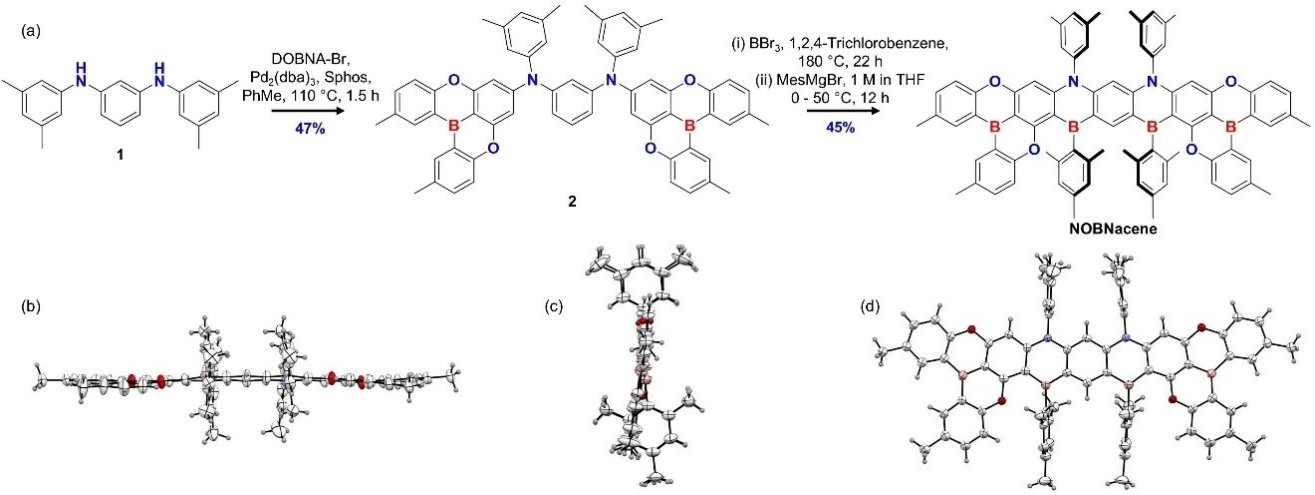
a) Synthesis of NOBNacene. ORTEP diagram of NOBNacene, b), c) are side views and d) is a plane view. Thermal ellipsoids show 50 % probability.

The convergent synthesis of NOBNacene is outlined in Figure 2. Compound 1 was obtained in 68 % yield following a Buchwald–Hartwig cross‐coupling reaction of 3,5‐dimethylaniline with 1,2‐dichlorobenzene. The key intermediate 2 was obtained in 47 % yield by coupling two equivalents of DOBNA‐Br with 1. Electrophilic borylation of 2 with BBr_3_ followed by reaction with excess MesMgBr afforded NOBNacene in 45 % yield. Due to its planar structure, NOBNacene is poorly soluble in most common organic solvents. The structure and purity of the emitter were confirmed by ^1^H NMR spectroscopy, high‐resolution mass spectrometry (HRMS), high performance liquid chromatography‐gel permeation chromatography (HPLC‐GPC), and elemental analysis (EA). The thermal behavior of NOBNacene was investigated by thermogravimetric analysis (TGA) and differential scanning calorimetry (DSC) (Figure S20). The compound is thermally stable, with 5 % mass loss (*T*_d_) occurring above 572 °C. A broad endothermic peak was observed in the DSC, with the onset temperature at 650 °C, corresponding to the material decomposition as supported by the TGA.

Single crystals of NOBNacene were obtained by slow evaporation of a saturated solution of the compound in THF over several days at room temperature.^[25]^ The crystal structure is shown in Figures 2b–d. No hydrogen bonding or π–π stacking intermolecular interactions were observed for NOBNacene in the crystal structure owing to the presence of the highly twisted mesityl and xylyl groups that decorate the nonacene core. As shown in the Figure 2b, the nonacene core of NOBNacene remains nearly planar. The B‐C_Mes_ bond is longer than other bonds in the system, because of this difference in bond lengths the nonacene skeleton is slightly bent as shown in Figure 2d.

**Figure 3:**
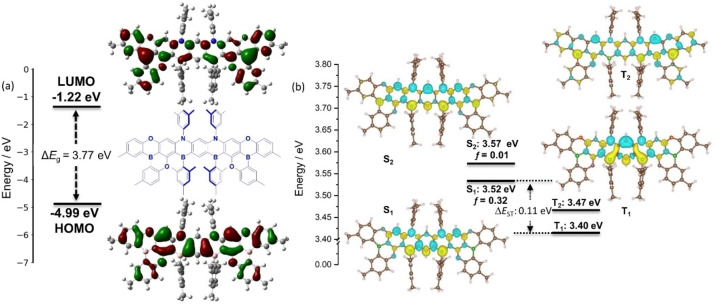
a) HOMO and LUMO electron density distribution and orbital energies of NOBNacene calculated at PBE0/6‐31G(d,p) in the gas phase (isovalue=0.02); b) Difference density plots and energies for the two lowest‐lying singlet and triplet excited states of NOBNacene calculated at SCS‐CC2/cc‐pVDZ in the gas phase (isovalue=0.001). The blue color represents an area of decreased electron density, and the yellow color represents an increased electron density between the ground and excited states. *f* denotes the oscillator strength for the transitions to the excited singlet states.

The ground [excited] state optimizations were carried out using [Time Dependent‐] Density Functional Theory [TD‐](DFT) with the PBE0 functional^[26]^ and the 6‐31G(d,p)^[27]^ basis set [within the Tamm‐Dancoff approximation (TDA)] in the gas phase while the excited states excitation energies were modelled with the spin‐component scaling second‐order approximate coupled‐cluster (SCS‐CC2) method. The latter wavefunction‐based method has been shown to be essential to model accurately the excited states of MR‐TADF emitters.^[28]^ In the ground state (Figure 3a), the HOMO is mainly localized on the atoms of the central benzene ring and the DOBNA fragments while the LUMO is mainly localized on the DOBNA boron atoms with a very small contribution from the mesityl boranes. The calculated HOMO and LUMO values are −4.99 eV and −1.22 eV, respectively, resulting in a predicted HOMO–LUMO gap of 3.77 eV. This gap is smaller than both DOBNA itself (3.97 eV)^[22]^ and the structurally related deep blue B,N‐doped heptacene derivatives, *α*‐3BNOH (4.20 eV) and *α*‐3BNMes (4.03 eV).[^18^, ^21^]

The difference density plots for the first singlet and triplet excited states show the alternating pattern of increasing and decreasing electron density that is characteristic of MR‐TADF emitters. The difference density plots reveal that the electron density in both the S_1_ and T_1_ states is mostly localized on the electron‐rich central part of the molecule. However, the difference density patterns of S_1_ and T_1_, while being reminiscent of SRCT excited states, are distinct from each other because of the different one‐electron transitions contributions (see Supporting Information, Tables S1 and S2). The electron density is more diffusely distributed across the entire molecular skeleton in the S_2_ and T_2_ states. A small Δ*E*_S1T1_ value of 0.12 eV is noted for NOBNacene (Table S1); however, owing to the different S_1_ and T_1_ difference density patterns, obtained from vertical excitations from the ground state, it is likely that the vertical Δ*E*_S1T1_ would not be accurate, as these excited states would exhibit different relaxation energies.^[29]^ We therefore undertook SCS‐CC2 calculations at the optimized S_1_ and T_1_ geometries and observed that the adiabatic Δ*E*_S1T1_ increases to 0.18 eV (Figure S11 and Table S2). An intermediate T_2_ state is reported between S_1_ and T_1_, obtained from vertical excitation from the ground state, which suggests that RISC could occur through a spin‐vibronic coupling mechanism. The S_0_−S_1_ transition has a high oscillator strength of 0.32 from calculations with vertical excitation from the ground state, which rises to 0.40 at the relaxed S_1_ geometry.

### Optoelectronic Characterization

**Figure 4:**
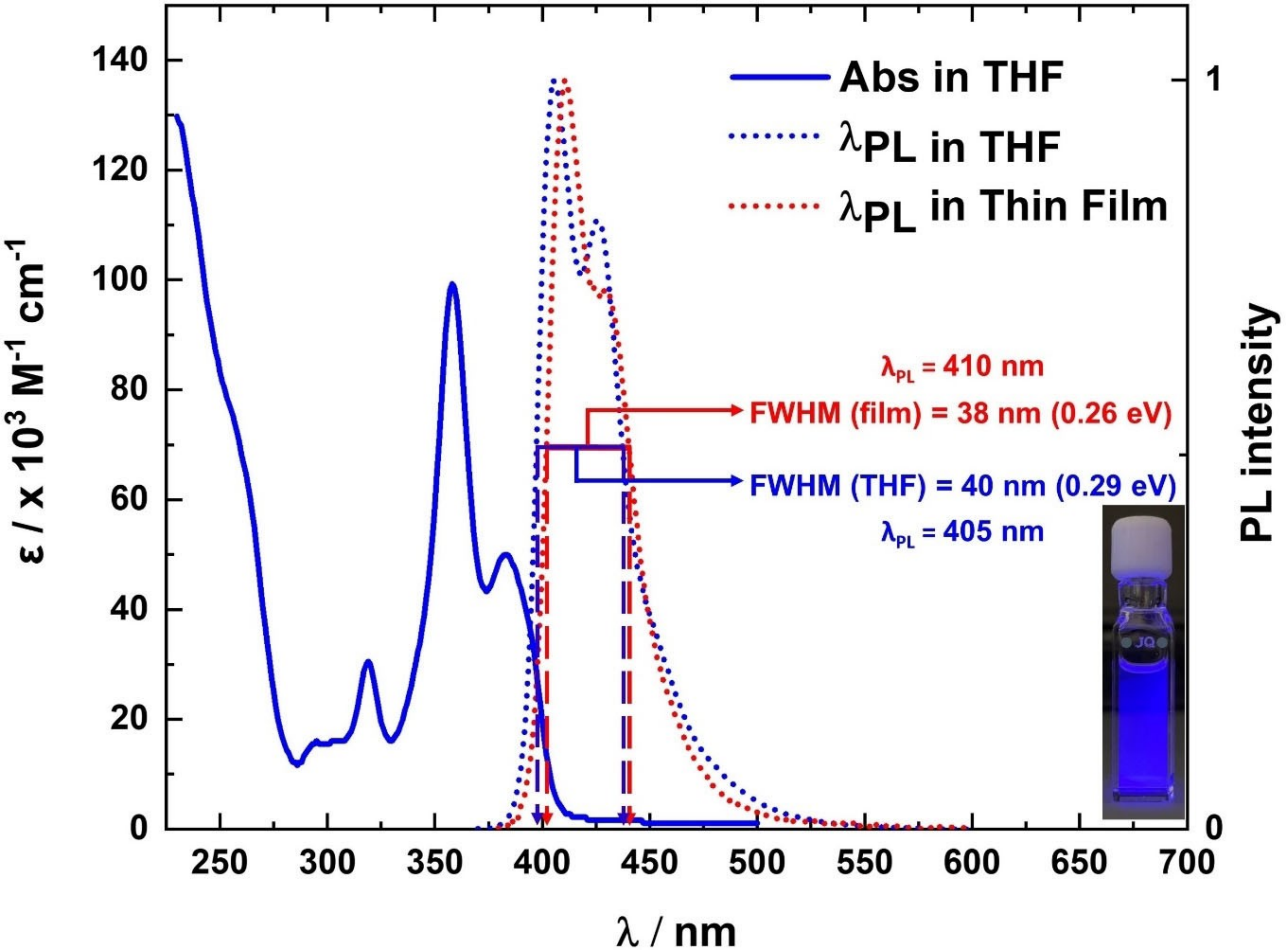
Absorption (blue solid line), steady‐state PL in THF at 300 K (blue dotted line), and steady‐state PL of the 1.5 wt % evaporated doped thin film in TSPO1 at 300 K (violet dotted line), *λ*_exc._=365 nm. Inset shows the PL of NOBNacene in THF at 300 K, *λ*_exc._=365 nm.

We first undertook a photophysical investigation of NOBNacene in dilute THF solutions (10^−5^ M) at 300 K (Figure 4 and Table 1), which would provide insight into understanding the monomolecular properties of this compound. An intense absorption was noted at 229–283 nm with molar absorptivity (*ϵ*) ranging from 130 to 12×10^3^ M^−1^ cm^−1^. A well‐defined, intense absorption band at 358 nm (*ϵ=*99×10^3^ M^−1^ cm^−1^) and a lowest energy band at 382 nm (*ϵ=*50×10^3^ M^−1^ cm^−1^) were observed corresponding to transitions mainly localized on the nonacene core as assigned from the TDA‐PBE0/6‐31G(d,p) calculations. The absorption profile is similar to that of α‐3BNOH^[21]^ and its derivative, α‐3BNMes.^[18]^ The lowest energy band in NOBNacene is slightly red‐shifted compared to that in α‐3BNOH (*λ*_abs_=379 nm, *ϵ=*14×10^3^ M^−1^ cm^−1^)^[21]^ but significantly blue‐shifted from that in *α*‐3BNMes (*λ*_abs_=419 nm, *ϵ=*18×10^3^ M^−1^ cm^−1^). The photoluminescence (PL) of NOBNacene in solution is structured and of intermediate broadness (FWHM of 40 nm, 0.29 eV) compared to other reported deep blue MR‐TADF emitters, see below.^[9]^ The emission maximum, *λ*_PL_, is at 405 nm and there is a low‐energy shoulder at 427 nm. We simulated the vibronically‐resolved emission spectrum within the undistorted harmonic model considering vibrational modes computed at the PBE0/6‐31G(d,p) level for the NOBNacene model derivative without the mesityl and xylyl substituents. We found that the broadening of the emission is essentially due to three vibrational modes with energies of 178, 643 and 1674 cm^−1^. The two lower‐frequency modes contribute to the main emission vibronic peak while the highest one leads to the side band (see Figures S13). The Δ*E*_ST_, calculated from the difference in the energy of the onsets of the prompt fluorescence and phosphorescence spectra at 77 K in 2‐MeTHF, is 0.31 eV (Figure S14a). This value is comparable to those of α‐3BNOH (Δ*E*_ST_=0.25 eV, 1 wt % in PMMA)^[21]^ and α‐3BNMes (Δ*E*_ST_=0.28 eV, 1 wt % in PMMA). The photoluminescence quantum yield (Φ_PL_) in degassed dilute THF solution is 33 %, which decreased under air to 25 %. No delayed emission was observed in THF and the lifetime, *τ*_PL_, is 22.4 ns (Figure S15).

**Table 1:** Photophysical properties of NOBNacene.

Compound	Medium	*λ*_PL_^[c]^ [nm]	FWHM^[d]^ [nm]	*E*_S1_^[e]^ [eV]	*E*_T1_^[e]^ [eV]	Δ*E*_ST_^[g]^ [eV]	Φ_PL_^[h]^ [%]	*τ*_p_^[i]^ [ns]	*τ*_d_^[i]^ [ms]	*k*_ISC_^[j]^ [s^−1^]	*k*_RISC_^[j]^ [s^−1^]	*k*_s_r_^[j]^ [s^−1^]	*k*_s_nr_^[k]^ [s^−1^]
NOBNacene	Sol.^[a]^	405 427	40	3.12^[f]^	2.81 ^[f]^	0.31	33^[a]^	22.34	–	–	–	–	–
	film^[b]^	410 430	38	3.12	2.82	0.30	71^[b]^	2.9	1.18	2.61×10^8^	3.74×10^3^	7.24×10^7^	1.18×10^7^

[a] In THF solutions (10^−5^ M). [b] Measured in evaporated thin films consisting of 1.5 wt % emitter in TSPO1 host under vacuum. *λ*_exc_=280 nm. [c] Steady‐state emission maximum at 300 K. *λ*_exc_=365 nm. [d] Full width at half maximum of the emission peak. [e] S_1_ and T_1_ energies were obtained from the onsets of the respective prompt fluorescence (delay: 1 ns; gate time: 100 ns) and phosphorescence spectra (delay: 1 ms; gate time: 9 ms) at 77 K. *λ*_exc_=343 nm. [f] 2‐MeTHF glass (10^−6^ M). [g] Δ*E*_ST_=*E*(S_1_)−*E*(T_1_). [h] Relative photoluminescence quantum yields (Φ_PL_) in solutions were measured by the relative method using quinine sulfate as a standard (Φ_r_=54.6 % in 1 N H_2_SO_4_).^[30]^ Absolute Φ_PL_ of thin films were measured using an integrating sphere. [i] ^*i*^Prompt and delayed lifetimes obtained by TCSPC and MCS, respectively. *λ*_exc_=379 nm. [j] Intersystem and reverse intersystem crossing rates were calculated using steady‐state approximation method as described in literature.^[31]^

We next explored the potential of this compound as an emitter in an OLED. Of the suitably high triplet energy hosts investigated (TSPO1, DPEPO, PPT, and mCP), the highest Φ_PL_ of 71 % was obtained when the emitter is doped in TSPO1 host at 1.5 wt %; the Φ_PL_ of this film reduced to 33 % upon exposure to air (see Table S3 for host matrix Φ_PL_ study), suggesting strongly that there are accessible triplet excited states. The PL spectrum of NOBNacene is red‐shifted by 5 nm in the 1.5 wt % TSPO1 doped film compared to in THF solution (Figure 4a). The FWHM of the emission spectrum in the doped film is 38 nm (0.26 eV). The high‐energy peak around 410 nm is the dominant one and there is a low‐energy shoulder at 430 nm, consistent with the theoretical calculations. The emission of NOBNacene is red‐shifted by 12 nm from DOBNA (*λ*_PL_=398 nm, Φ_PL_=58 %, in 1 wt % PMMA).^[22]^ The Δ*E*_ST_ is 0.30 eV in the 1.5 wt % TSPO1 thin film, a value that is nearly identical to that observed in 2‐MeTHF (Figure S14).

**Figure 5:**
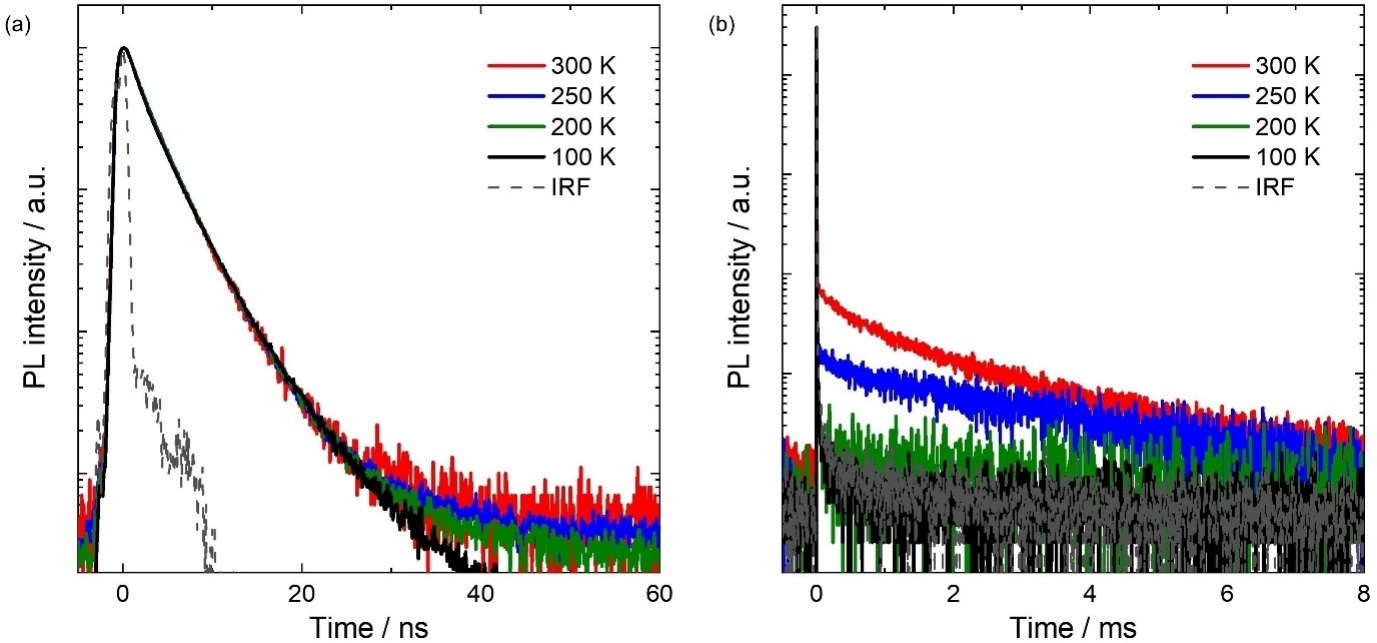
Time‐resolved PL decays of NOBNacene doped thin films (c_D_=1.5 wt % in TSPO1 host) under various temperatures from 300 K to 100 K. The measurements were performed by using TCSPC with samples loaded in a cryostat in vacuum. a) TCSPC at a 100 ns time scale, b) MCS at a 10 ms time scale. IRF is instrumental response function. The excitation wavelength was 379 nm and the PL emission at 410 nm was recorded.

Temperature‐dependent transient PL analysis was carried out to identify the nature of the triplet harvesting in NOBNacene. We observed a biexponential emission decay with associated prompt (*τ*_p_) and delayed (*τ*_d_) lifetimes of 2.90 ns and 1.18 ms, respectively (Figures 5a and b). The prompt component shows no temperature dependence, while the intensity of the delayed emission increases above 250 K. The temperature dependence of the delayed emission provides confirmation that NOBNacene is TADF active. TADF in emitters with large Δ*E*_ST_ have previously been reported by Lu et al.^[32]^ in a D‐A type violet emitter CZ‐MPS that exhibited a very long *τ*_d_ of 4.85 ms linked to a Δ*E*_ST_ of 0.58 eV. The authors ascribed the TADF to proceeding via intermediate triplet states. The *k*_RISC_ value of NOBNacene was calculated to be 3.74×10^3^ s^−1^, which is slower than what reported for DOBNA (1.6×10^4^ s^−1^, in 1 wt % PMMA),^[22]^ but faster than for α‐3BNMes (5.9×10^2^ s^−1^, in 1 wt % PMMA).^[18]^

Given the high‐energy emission, the choice of host matrix with suitable high triplet energies is very limited. From the photophysical study TSPO1 was identified as the most suitable host matrix to be used in the OLED. The optimized device structure used was: ITO/HAT‐CN (5 nm)/NPB (40 nm)/TCTA (10 nm)/CzSi (10 nm)/NOBNacene (1.5–6.0 wt %) : TSPO1 (20 nm)/TSPO1 (10 nm)/TmPyPB (20 nm)/LiF (0.8 nm)/Al (100 nm), where HAT‐CN, NPB, TCTA, CzSi, TSPO1, TmPyPB, LiF are the hole injection, hole transporting, electron blocking, exciton blocking, hole blocking, electron transporting, and electron injection layers, respectively. The device structure and energy levels of each layer are illustrated in Figure S17. OLED devices were fabricated using c_D_ of 1.5 wt %, 3.0 wt %, and 6.0 wt % to understand its effects on both carrier transport and color purity. Device performance metrics are summarized in Table 2.

**Table 2:** Device data of NOBNacene in TSPO1 and DPEPO host.

Host	c_D_ [wt %]	V_on_ [V]	*λ*_EL_ [nm]	FWHM/nm	CIE (*x*,*y*)	EQE_max_ [%]
TSPO1	1.5 3.0 6.0	4.2 4.2 4.1	409 412 412	37 41 47	(0.173, 0.055) (0.176, 0.068) (0.187, 0.103)	8.5 11.2 10.2
DPEPO	1.5	5.1	411	44	(0.196, 0.106)	4.2

The device with c_D_ of 1.5 wt % in TSPO1 host presented narrow violet emission with a maximum at *λ*_EL_ of 411 nm (FWHM=37 nm) and a shoulder at 428 nm, which are assigned as vibronic bands. The EL matches well the PL of the film at the same c_D_ (*λ*_PL_=410 nm, FWHM=38 nm, 1.5 wt % in TSPO1). At 3 wt % doping concentration, the *λ*_EL_ is nearly unchanged at 412 nm compared to the EL of the 1.5 wt % device; however, the FWHM increased slightly to 41 nm and the shoulder at 428 nm is more pronounced. The *λ*_EL_ of the device with c_D_ of 6.0 wt % is essentially the same (412 nm); however, there is significant broadening of the EL (FWHM=47 nm) due to the more pronounced shoulder at 428 nm. The broadening of emission for fused planar emitters at a higher concentration is common due to intermolecular interaction in the emitting layers.^[33]^ Due to its high E_T_, we also fabricated devices using DPEPO as the host at the same c_D_ of 1.5 wt % for comparison purposes. Besides the lower EQE, the EL spectrum is broadened (FWHM=44 nm) and the turn‐on voltage is higher by 0.9 V in the DPEPO device compared to that in TSPO1. In addition, the device showed incomplete electron confinement within the EML as evidenced by the high‐energy EL peak at 380 nm, which was assigned to emission from the TCTA layer.

**Figure 6:**
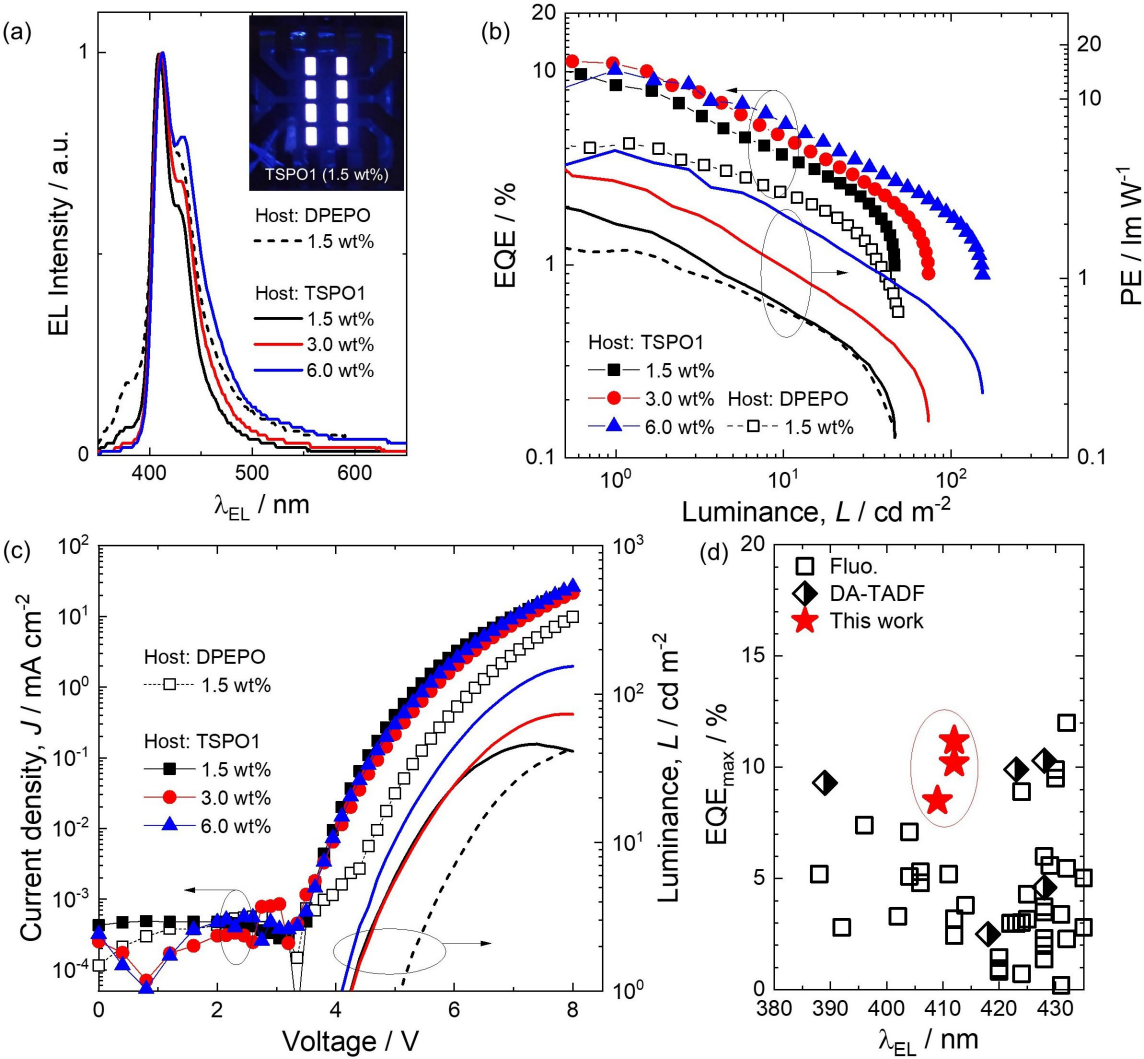
OLED device performance for NOBNacene in both DPEPO and TSPO1 host. a) EL spectrum of devices at 6 V. Inset shows Image of an operating TSPO1 device (*c*_D_=1.5 wt %) under 6 V. b) Dependence of external quantum efficiency (EQE) and power efficiency (PE) on the luminance. c) Current density‐voltage‐luminance (*J*–*V*–L) characteristics of the device. d) EQE_max_ comparison in terms of EL peak wavelength to reported fluorescent (Fluo.) and D‐A type TADF emitters. source references for the plotted data are given in Table S4 and compared with literature devices.

The CIE coordinates of the TSPO1 devices with c_D_ of 1.5 wt % are (0.173, 0.055), values that are very close to the Rec.2020 standard for primary blue in UHDTV (0.131, 0.046).^[24]^ Increasing the doping concentration of the emitter resulted in the expected reduced colour purity of the device, reflected in the CIE coordinates of (0.176, 0.068) and (0.187, 0.103), respectively, for the 3 wt % and 6 wt % devices. The EQE_max_ (PE_max_) were 8.5 % (2.06 lm W^−1^), 11.2 % (3.51 lm W^−1^) and 10.2 % (5.19 lm W^−1^) for devices with c_D_ of 1.5 wt %, 3.0 wt % to 6.0 wt %, respectively. The high EQE (11.2 %) of the device at this deep blue chromaticity (CIEy<0.08) is among the highest recorded to date (Figure 6d). The device data are summarized in Table S4. A linear dependence of luminescence to current density (Figure S18) and the presence of delayed component in the transient PL analysis provide strong indications that triplet harvesting in the device occurs through TADF. Despite the high EQE_max_, the efficiency roll‐off in each system is severe, which is likely due in part to the large Δ*E*_ST_ of the emitter,^[33]^ the long delayed lifetimes that result in increased TTA and STA processes, and the instability of the phosphine oxide‐based host.^[34]^ The EL spectra were unchanged during operation, while the intensity decreased rapidly to 50 % of its initial value within 10 min. Such behavior is not uncommon in deep blue OLEDs, especially considering the electrically unstable phosphine oxide‐based TSPO1 host.

## Conclusion

Herein, we demonstrate an easy‐to‐access synthetic route to construct a boron, nitrogen, and oxygen doped ladder type nonacene. The emitter NOBNacene is the first example of a π‐extended MR‐TADF emitter that has nine, six‐membered rings fused along one dimension. Strikingly, this π‐extended design shows only a limited conjugation length, reflected in emission in the deep blue region in THF solution (*λ*_PL_=405 nm, FWHM=40 nm, Φ_PL_=33 %) and in the 1.5 wt % TSPO1 film (*λ*_PL_=410 nm, FWHM=38 nm, Φ_PL_=71 %, *τ*_d_=1.77 ms). This emitter design utilizes two electronically distinct boron acceptor atoms and a combination of nitrogen and oxygen donor atoms. Deep blue EL (*λ*_EL_=409 nm, FWHM=37 nm) was produced when NOBNacene was employed as the emitter in the OLED. An EQE_max_ of 8.5 % at CIE coordinates of (0.173, 0.055) was achieved. The EQE_max_ could be enhanced to 11.3 % at a higher emitter doping of 3 wt %, with only a small loss in the color purity, reflected in CIE coordinates of (0.176, 0.068). The EQE_max_ values reported here are among the highest reported for deep‐blue OLED devices where the *λ*_EL_<420 nm. Compared with the reported fluorescent, donor‐acceptor type TADF, and MR‐TADF based OLEDs shown in Figure 6d, the NOBNacene device combines near UV emission at 410 nm, high EQE_max_ of over 10 %, and deep blue color purity with CIEy of 0.06, a device performance that is promising not only for displays but also for applications that require a near UV light source.

## Conflict of interest

The authors declare no conflict of interest.

1

## Supporting information

As a service to our authors and readers, this journal provides supporting information supplied by the authors. Such materials are peer reviewed and may be re‐organized for online delivery, but are not copy‐edited or typeset. Technical support issues arising from supporting information (other than missing files) should be addressed to the authors.

Supporting Information

Supporting Information

## Data Availability

The research data supporting this publication can be accessed at https://doi.org/10.17630/bbf0fb10‐6225‐4422‐8b0b‐73c965e2e6ba.
