# Supplementary material for: A Deep‐Blue‐Emitting Heteroatom‐Doped MR‐TADF Nonacene for High‐Performance Organic Light‐Emitting Diodes
Source: Angew Chem Int Ed Engl. 2023 Jan 16;62(8):e202215522. doi: 10.1002/anie.202215522 (PMC10107802; doi:10.1002/anie.202215522)
Supplement: Supplementary file 1 — Supporting Information [file ANIE-62-0-s002.pdf]

Angewandte Chemie
Eine Zeitschrift der Gesellschaft Deutscher Chemiker

Supporting Information

A Deep-Blue-Emitting Heteroatom-Doped MR-TADF Nonacene for High-Performance Organic Light-Emitting Diodes

S. Madayanad Suresh, L. Zhang, D. Hall, C. Si, G. Ricci, T. Matulaitis, A. M. Z. Slawin, S. Warriner, Y. Olivier, I. D. W. Samuel, E. Zysman-Colman**

SUPPORTING INFORMATION

Table of Contents

	Page
**General Methods**	**S2**
**Experimental Section**	**S7**
**^{1}H, ^{13}C NMR spectra and HRMS**	**S10**
**Supplementary Calculations**	**S15**
**Photophysical Characterization**	**S17**
**Device Characterization**	**S21**
**Literature study**	**S23**
**TGA/DSC**	**S24**
**References**	**S26**

Experimental Section

General Synthetic Procedures. All other reagents and solvents were obtained from commercial sources and used as received. Air-sensitive reactions were performed under a nitrogen atmosphere using Schlenk techniques, no special precautions were taken to exclude air or moisture during work-up and crystallisation. Anhydrous THF, DCM, and toluene were obtained from a MBraun SPS5 solvent purification system. Flash column chromatography was carried out using silica gel (Silia-P from Silicycle, 60 Å, 40-63 μm). Analytical thin-layer-chromatography (TLC) was performed with silica plates with aluminum backings (250 μm with F-254 indicator). TLC visualization was accomplished by 254/365 nm UV lamp. HPLC analysis was conducted on a Shimadzu LC-40 HPLC system. GCMS analysis was conducted using a Shimadzu QP2010SE GC-MS equipped with a Shimadzu SH-Rtx-1 column (30 m × 0.25 mm). ^{1}H, and ^{13}C NMR spectra were recorded on a Bruker Advance spectrometer (500 or 700 MHz for ^{1}H NMR, 125 or 175 MHz for ^{13}C NMR). The following abbreviations have been used for multiplicity assignments: "s" for singlet, "d" for doublet, and "t" for triplet. ^{1}H and ^{13}C NMR spectra were referenced residual solvent peaks with respect to TMS (δ = 0 ppm). Melting points were measured using open-ended capillaries on an Electrothermal 1101D Mel-Temp apparatus and are uncorrected. High-resolution mass spectrometry (HRMS) was performed by University of Edinburgh and University of Leeds. Elemental analysis was performed by School of Geosciences at the University of Edinburgh.

Photophysical measurements. Optically dilute solutions of concentrations on the order of 10^{-5} to 10^{-6} M were prepared in spectroscopic or HPLC grade solvents for absorption and emission analyses. Absorption spectra were recorded at room temperature on a Shimadzu UV-2600 double beam spectrophotometer with a 1 cm quartz cuvette. Molar absorptivity determination was verified by linear regression analysis of values obtained from at least five independent solutions at varying concentrations ranging from 10^{-5} M to 10^{-6} M. Steady-state emission and excitation spectra and time-resolved emission decays were recorded at 298 K using an Edinburgh Instruments FS5 spectrofluorometer. Samples were excited at 378 nm for steady-state measurements and at 378 nm for time-resolved measurements.

For photoluminescence quantum yield measurements, degassed solutions were prepared via three freeze-pump-thaw cycles and spectra were taken using home-made Schlenk quartz cuvette. Photoluminescence quantum yields for solutions were determined using the optically dilute method[1] in which four sample solutions with absorbances of ca. 0.19, 0.15, 0.09 and 0.05 for **NOBNacene**. The Beer-Lambert law was found to remain linear at the concentrations of the solutions. For each sample, linearity between absorption and emission intensity was verified through linear regression analysis with the Pearson regression factor (R^2) for the linear fit of the data set surpassing 0.9. Individual relative quantum yield values were calculated for each solution and the values reported represent the slope obtained from the linear fit of these results. The quantum yield of the sample, Φ_{PL}, can be determined by the equation $\Phi_{PL} = (\Phi_r * \frac{A_r}{A_s} * \frac{I_s}{I_r} * \frac{n_s^2}{n_r^2})^2$, where *A* stands for the absorbance at the excitation wavelength (λ_{exc} = 378 nm), *I* is the integrated area under the corrected emission curve and *n* is the refractive index of the solvent with the subscripts "s" and "r" representing sample and reference respectively. Φ_r is the absolute quantum yield of the external reference quinine sulfate (Φ_r = 54.6% in 1 N H_2SO_4),[2] The experimental uncertainty in the emission quantum yields is conservatively estimated to be 10%, though we have found that statistically we can reproduce Φ_{PL} values to 3% relative error.

Evaporated thin films were used to measure thin film photoluminescence properties in the solid state. An integrating sphere (Hamamatsu, C9920-02) was employed for photoluminescence quantum yield measurements for thin film samples. Time-resolved PL measurements of the thin films were carried out using the multi-channel scaling (MCS) technique and time correlated single photon counting system (TCSPC). The film samples were excited at 378 nm by a pulsed laser diode (Picoquant, model PLS 370) and were kept in a vacuum of $< 8 \times 10^{-4}$ mbar.

Prompt fluorescence and phosphorescence spectra were obtained from dilute solutions of samples in 2-methyltetrahydrofuran and evaporated films at 77 K and the singlet-triplet splitting energy gap, ΔE_{ST}, was estimated from the onsets of the corresponding spectra. For ΔE_{ST} measurements, film samples were loaded

inside a cold finger cryostat (Oxford Instruments) and placed under vacuum, with temperature controlled from 300 K – 77 K, while an open Dewar filled with liquid nitrogen was used for solution samples. All samples were photoexcited using the third harmonic emission (343 nm) from a femtosecond Nd:YAG laser, which originally emits at 1030 nm (Orpheus-N, model: PN13F1). Emission from the samples was focused onto a spectrograph (Chromex imaging, 250is spectrograph) and detected on a sensitive gated iCCD camera (Stanford Computer Optics, 4Picos) having subnanosecond resolution. Phosphorescence spectra were measured 1 ms after the excitation of the Nd:YAG laser with iCCD exposure time of 9 ms. Prompt fluorescence spectra were measured 1 ns after the excitation of the femtosecond laser with iCCD exposure time of 100 ns.

Fitting of time-resolved luminescence measurements: Time-resolved PL measurements were fitted to a sum of exponentials decay model, with chi-squared (χ^2) values between 1 and 2, using the EI software. Each component of the decay is assigned a weight, (w_i), which is the contribution of the emission from each component to the total emission.

The average lifetime was then calculated using the following:

- Two exponential decay model:

$$\tau_{AVG} = \tau_1 w_1 + \tau_2 w_2$$

with weights defined as $w_1 = \frac{A1\tau_1}{A1\tau_1 + A2\tau_2}$ and $w_2 = \frac{A2\tau_2}{A1\tau_1 + A2\tau_2}$ where A1 and A2 are the preexponential-factors of each component.

- Three exponential decay model:

$$\tau_{AVG} = \tau_1 w_1 + \tau_2 w_2 + \tau_3 w_3$$

with weights defined as $w_1 = \frac{A1\tau_1}{A1\tau_1 + A2\tau_2 + A3\tau_3}$, $w_2 = \frac{A2\tau_2}{A1\tau_1 + A2\tau_2 + A3\tau_3}$ and $w_3 = \frac{A3\tau_3}{A1\tau_1 + A2\tau_2 + A3\tau_3}$ where A1, A2 and A3 are the preexponential-factors of each component.

Quantum chemical calculations. The calculations were performed with the Gaussian 16 revision A03 suite[3] for the density functional theory (DFT) and with the Turbomole 7.4 package for SCS-CC2

calculations. We optimized the ground state using the PBE0 functional[4] with the 6-31G(d,p)[5] basis set and the S_1 and T_1 excited states using TDA-PBE0/6-31G(d,p). Excited state calculations from the ground state geometry were performed based on the ground-state-optimized structure using spin-component scaling coupled-cluster singles-and-doubles model (SCS-CC2) with the cc-pVDZ basis set.[6] computing the two lowest-lying singlet (S_1 and S_2)[7] and two lowest-lying triplet excited states (T_1 and T_2).[8] Excited-state calculations were performed on the S_1 and T_1 optimized geometries at the SCS-CC2/cc-pVDZ level of theory. UV-Vis absorption spectra were simulated by calculating the first 20 singlet excited states at the TDA-PBE0/6-31G(d,p) level, with a peak broadening of 0.05 eV. The difference density plots obtained at the TDA-DFT were calculated as the difference between the attachment and detachment densities computed with the NANCY package.[9] Molecular orbitals were visualized using GaussView 6.0.[10] Difference density plots were used to visualize change in electronic density between the ground and excited state and were obtained using the VESTA package.[11]

X-ray crystallography. Crystals for **NOBNacene** were obtained by a slow evaporation of saturated solution of THF at room temperature over several days.

EXPERIMENTAL DETAILS

A. Crystal Data

Empirical Formula	$C_{80}H_{68}B_4N_2O_4$
Formula Weight	1164.67
Crystal Color, Habit	colourless, plate
Crystal Dimensions	0.050 × 0.030 × 0.010 mm
Crystal System	triclinic
Lattice Type	Primitive
Lattice Parameters	a = 7.8873(5) Å
	b = 16.2364(10) Å
	c = 25.5485(12) Å
	α = 75.657(5)°
	β = 85.804(5)°
	γ = 82.774(5)°
	V = 3141.6(3) $Å^3$
Space Group	P-1 (#2)
Z value	2

D_{calc}	1.231 g/cm^3
F_{000}	1228.00
μ(CuKα)	5.713 cm^{-1}

B. Intensity Measurements

Diffractometer	XtaLAB P100
Radiation	CuKα (λ = 1.54184 Å)<br>multi-layer mirror monochromated
Temperature	-100.0 °C
Detector Aperture	83.8 × 33.5 mm
Data Images	3982 exposures
Pixel Size	0.172 mm
$2\theta_{max}$	136.3°
No. of Reflections Measured	Total: 33171<br>Unique: 11199 (R_{int} = 0.1272)
Corrections	Lorentz-polarization<br>Absorption<br>(trans. factors: 0.255 - 0.994)

C. Structure Solution and Refinement

Structure Solution	Direct Methods (SHELXT Version 2018/2)
Refinement	Full-matrix least-squares on F^2
Function Minimized	$\Sigma w (Fo^2 - Fc^2)^2$
Least Squares Weights	$w = 1/ [\sigma^2(Fo^2) + (0.1129 \cdot P)^2 + 0.0000 \cdot P]$<br>where $P = (Max(Fo^2,0) + 2Fc^2)/3$
$2\theta_{max}$ cutoff	136.3°
Anomalous Dispersion	All non-hydrogen atoms
No. Observations (All reflections)	11199
No. Variables	825
Reflection/Parameter Ratio	13.57
Residuals: R1 (I>2.00σ(I))	0.0793
Residuals: R (All reflections)	0.1614
Residuals: wR2 (All reflections)	0.2310
Goodness of Fit Indicator	0.957
Max Shift/Error in Final Cycle	0.000
Maximum peak in Final Diff. Map	0.35 $e^-/Å^3$
Minimum peak in Final Diff. Map	-0.37 $e^-/Å^3$

Synthesis

*N*1,*N*3-bis(3,5-dimethylphenyl)benzene-1,3-diamine (**1**)

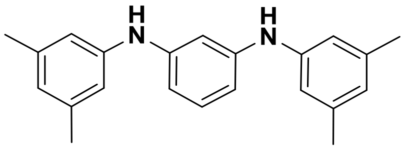

To a Schlenk flask under nitrogen was added, 1,3-dichlorobenzene (1.55 mL, 13.6 mmol, 1.0 equiv.), 3,5-dimethylaniline (5.09mL, 40.8 mmol, 3.0 equiv.), Sodium *tert*-butoxide (7.84 g, 81.6 mmol, 6.0 equiv.), Palladium acetate (0.092 g, 0.408 mmol, 0.03 equiv.), tri-*tert* butyl phosphonium tetrafluoroborate (0.166 g, 0.572 mmol, 0.042 equiv.) and 25 mL toluene. After stirring at 110 °C for 3 days, the reaction mixture was brought to RT and extracted with 200 mL ethyl acetate. This is then washed with DI water (100 mL × 3), the organic layer was dried in sodium sulfate. The crude mixture was purified by silica gel flash column chromatography using EtOAc : hexane = 6 : 94. The corresponding fractions were combined and the solvents were removed under reduced pressure, the product was obtained as white solid after dried under high vacuum overnight. **Yield**: 68%. **R_f:** 0.46 (EtOAc : hexane = 15:85 on silica gel). **Mp**: 90-93 °C. **^{1}H NMR (500 MHz, DMSO-d6) δ** 7.89 (s, 2H), 7.05 (t, *J* = 8.0 Hz, 1H), 6.78 (t, *J* = 2.0 Hz, 1H), 6.68 (s, 4H), 6.50 (dd, *J* = 8.0, 2.1 Hz, 2H), 6.46 (s, 2H), 2.20 (s, 12H). **^{13}C NMR (100 MHz, DMSO) δ** 144.94, 143.91, 138.41, 130.08, 121.83, 115.37, 109.12, 105.33, 21.67. **GC-MS[M]$^+$:** Calculated: ($C_{22}H_{24}N_2$) 316.19; Found: 316.20.

N1,N3-bis(2,12-dimethyl-5,9-dioxa-13b-boranaphtho[3,2,1-de]anthracen-7-yl)-N1,N3-bis(3,5-dimethylphenyl)benzene-1,3-diamine (2)

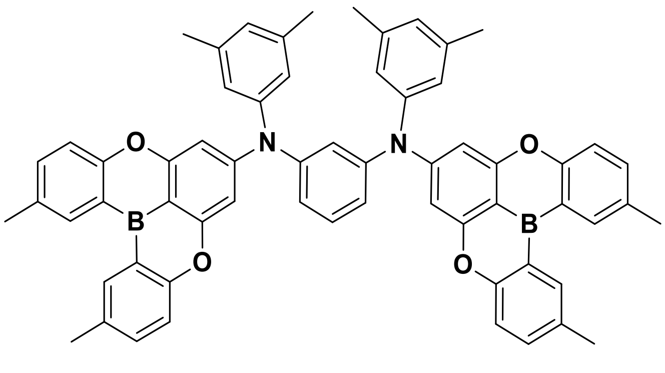

To a Schlenk flask under nitrogen was added, Compound **1** (0.79 g, 2.5 mmol, 1.0 equiv.), Sodium *tert*-butoxide (2.4 g, 25.0 mmol, 10.0 equiv.), $Pd_2(dba)_3$ (0.069 g, 0.075 mmol, 0.03 equiv.), Sphos (0.062 g, 0.15 mmol, 0.06 equiv.) and 40 mL toluene, **DOBNA-Br** (3.30 g, 8.75 mmol, 3.5 equiv.) was added to the reaction mixture under a positive flow of nitrogen. After stirring at 110 °C for 1.5 h, the reaction mixture was brought to RT and extracted with 200 mL DCM. This is then washed with DI water (100 mL × 3). The crude mixture was purified by silica gel flash column chromatography using DCM : hexane = 20 : 80 to DCM : hexane = 80 : 20 gradient. The corresponding fractions were combined and concentrated under reduced pressure to afford a greenish white solid, which was sonicated for 5 min, cooled at -20 °C in a freezer overnight and filtered. **Yield**: 47%. **R_f:** 0.56 (EtOAc : hexane = 15:85 on silica gel). **Mp**: 296-300 °C. **^{1}H NMR (500 MHz, CD_2Cl_2) δ** 8.46 (s, 4H), 7.49 (dd, *J* = 8.5, 1.8 Hz, 4H), 7.34 (m, 5H), 7.18 (t, *J* = 2.1 Hz, 1H), 7.06 (dd, *J* = 8.0, 2.1 Hz, 2H), 6.93 (s, 4H), 6.87 (s, 2H), 6.74 (s, 4H), 2.56 (s, 12H), 2.31 (s, 12H). **^{13}C NMR (176 MHz, CD_2Cl_2) δ** 159.26, 159.02, 154.66, 148.43, 146.70, 140.06, 134.68, 132.47, 130.93, 127.53, 124.98, 124.17, 122.59, 118.29, 100.50, 21.60, 21.35. **HR-MS[M]$^+$:** Calculated: ($C_{62}H_{50}B_2N_2O_4$) 908.3970; Found: 908.3984

NOBNacene

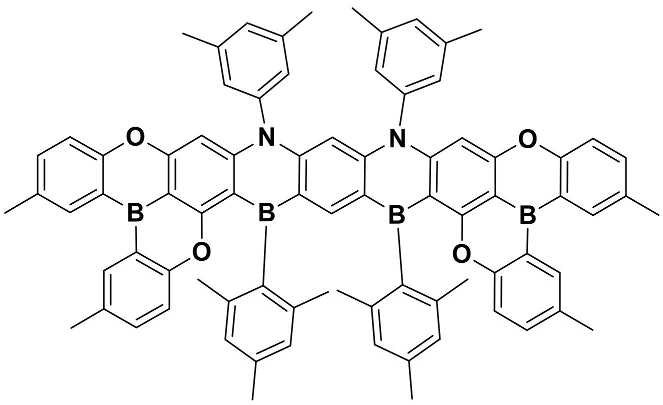

A Schlenk flask was charged with Compound **2** (0.35 g, 0.385 mmol, 1 equiv.) under nitrogen and dissolved in 5 mL anhydrous 1,2,4-trichlorobenzene. To this solution, BBr_3 (364 μL, 3.85 mmol, 10 equiv.) was added dropwise at RT. After stirring at 180 ºC for 22 h, excess BBr_3 was distilled off under a positive flow of nitrogen at 180 ºC for 1.5 h. The reaction mixture was cooled to 0 ºC and dropwise added 2-

mesitylmagnesium bromide (1 M sol. in THF, 1.32 mL, 15.0 mmol, 15.0 equiv.). After stirring at 50 ºC for 12 h, the reaction mixture was diluted with 200 mL of DCM and 100 mL water. The organic layer was separated, and the aqueous layer was washed several times with DCM to recover all the solids in the water layer. The organic layer was combined and concentrated and mixed with 100 mL THF and sonicated for 15 min. A white solid formed and was filtered and dried. **Yield**: 45%. **Mp**: No melting point observed below 400 ºC. **^{1}H NMR (700 MHz, CD_2Cl_2) δ** 8.43 (s, 2H), 8.39 (s, 2H), 8.27 (s, 1H), 7.45 (d, *J* = 9.2 Hz, 2H), 7.34 (dd, *J* = 8.4, 1.6 Hz, 2H), 7.27 (d, *J* = 8.5 Hz, 2H), 7.23 (s, 2H), 6.88 (s, 8H), 6.51 (s, 2H), 6.26 (d, *J* = 8.4 Hz, 2H), 5.86 (s, 1H), 2.51 (s, 6H), 2.49 (s, 12H), 2.41 (s, 12H), 1.96 (s, 12H). Carbon NMR was not recorded due to poor solubility. **HPLC-GPC** purity: 100%. Retention time: 10.235 mint. **HR-MS[M+H]$^+$:** Calculated: ($C_{80}H_{69}B_4N_2O_4$) 1165.5665; Found: 1165.5657. **Anal. Calcd**. for $C_{80}H_{68}B_4N_2O_4$: C, 82.50%; H, 5.89%; N, 2.41%. **Found** (average of 3 measurements) C, 82.89%; H, 5.99%; N, 2.50.

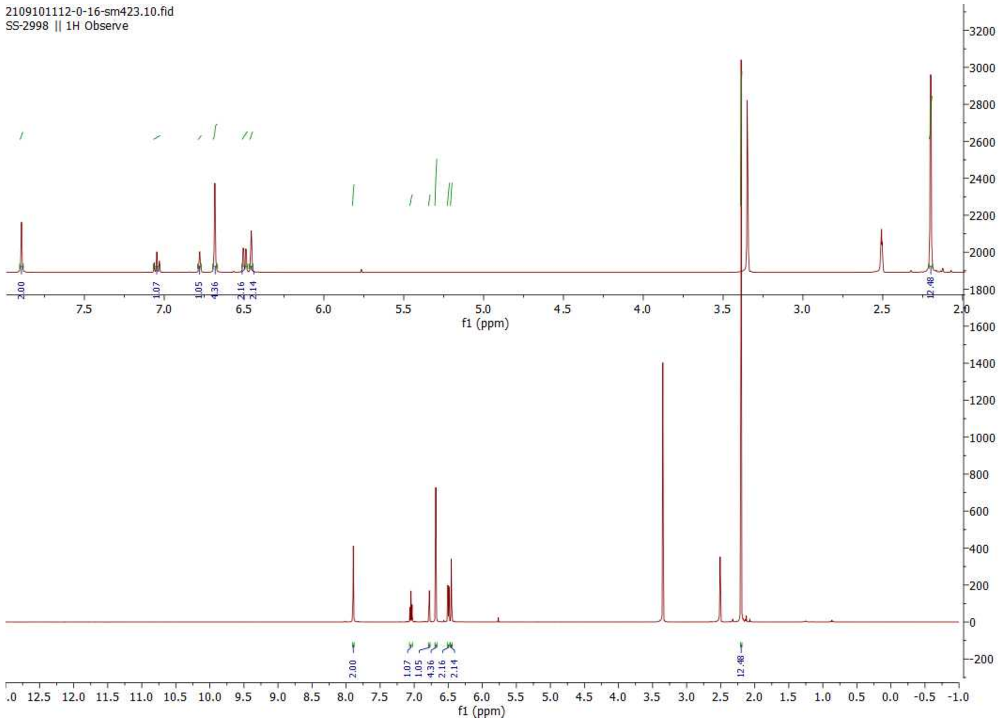

Figure **S1**. ^{1}H NMR of **1** in DMSO-d_6.

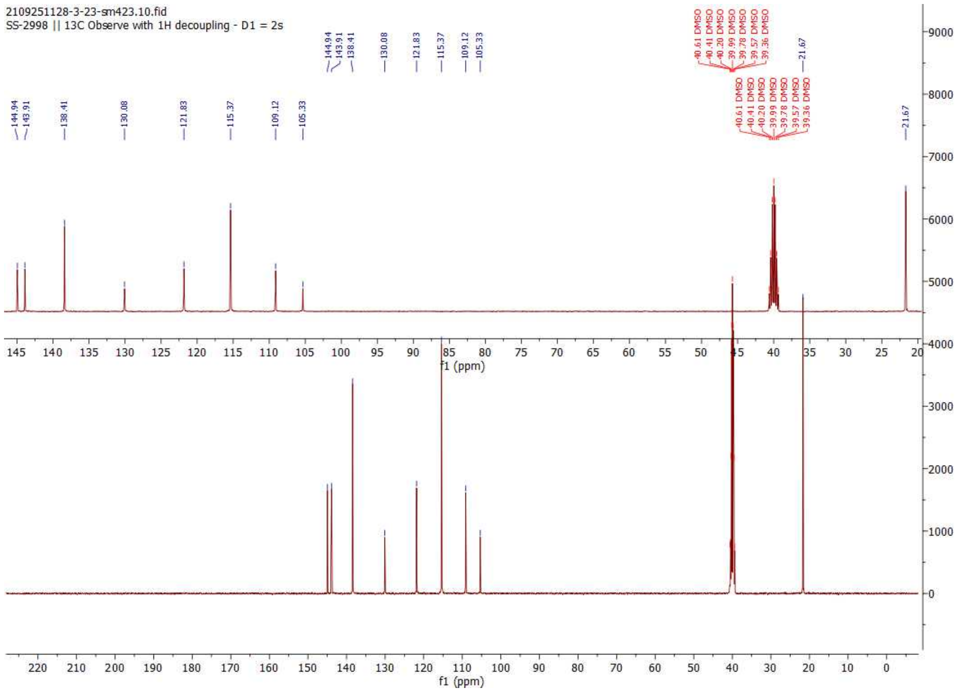

Figure **S2**. ^{13}C NMR of **1** in DMSO-d_6.

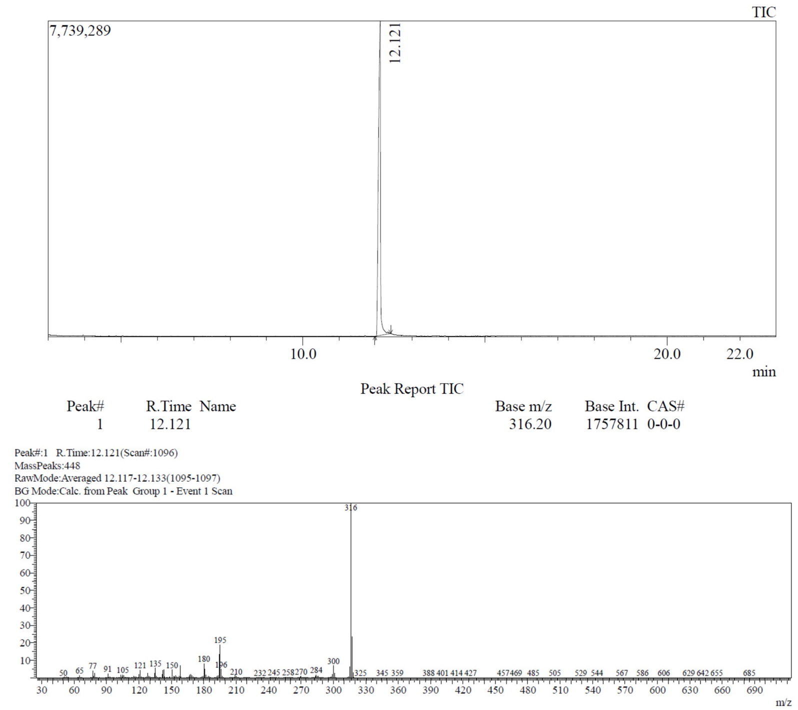

Figure **S3**. GCMS of **1**.

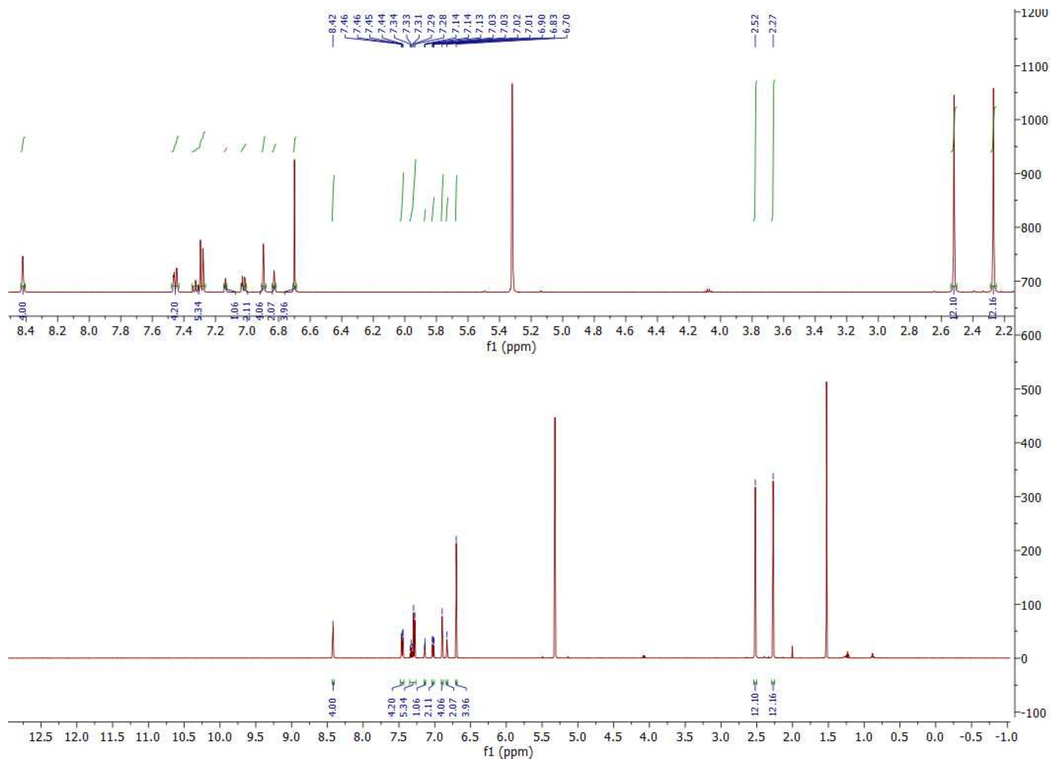

Figure **S4**. ^{1}H NMR of **2** in CD_2Cl_2.

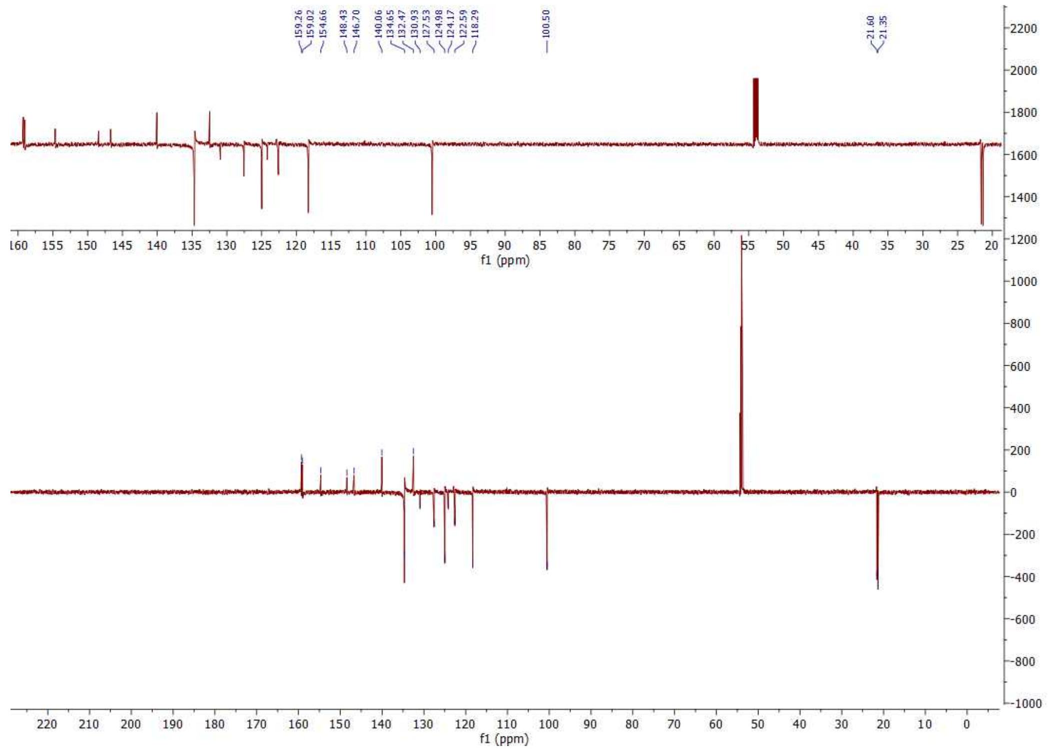

Figure **S5**. ^{13}C NMR of **2** in CD_2Cl_2.

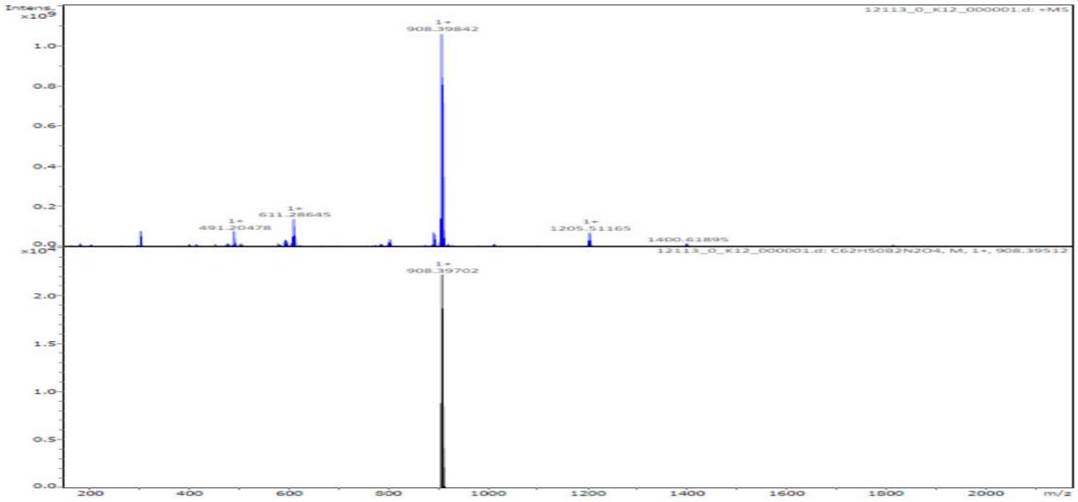

Figure **S6**. HRMS of **2**.

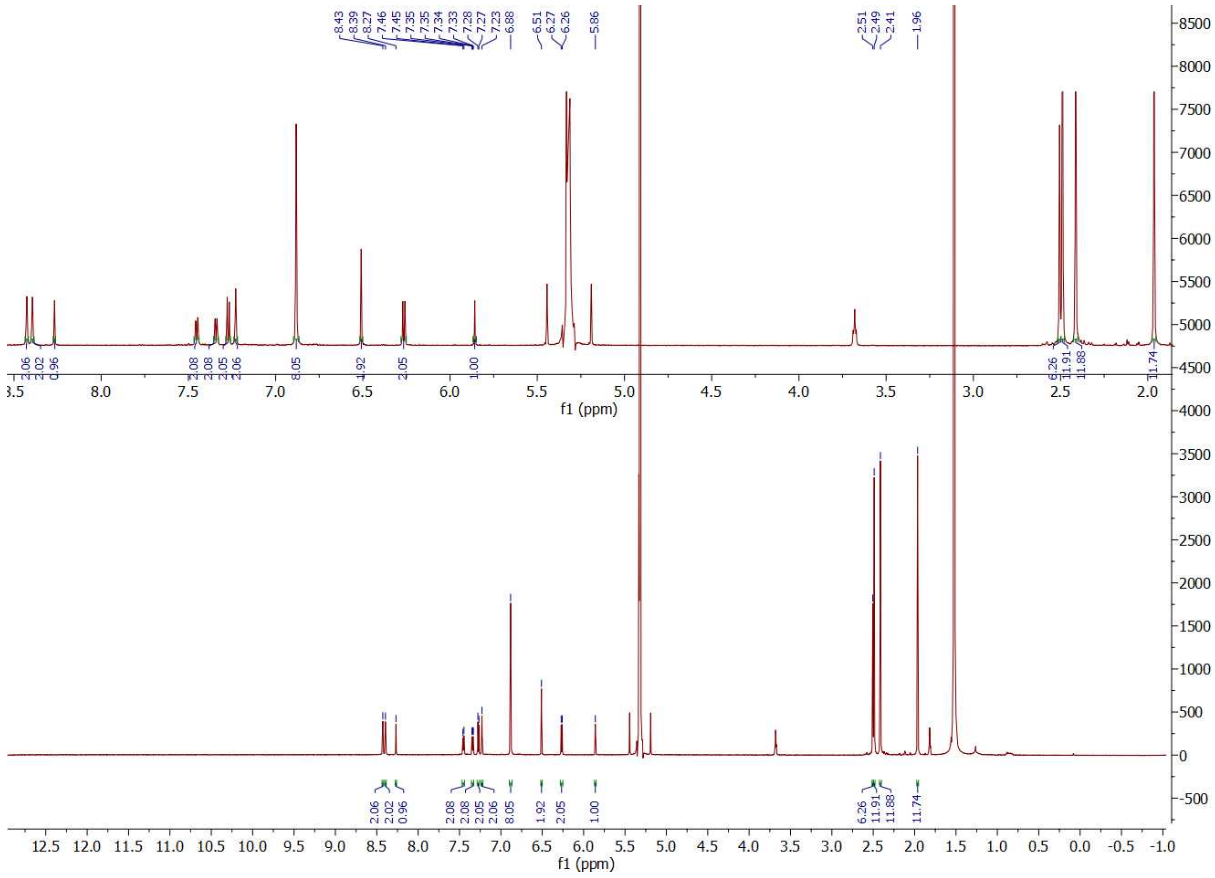

Figure **S7**. ^{1}H NMR of **NOBNacene** in CD_2Cl_2.

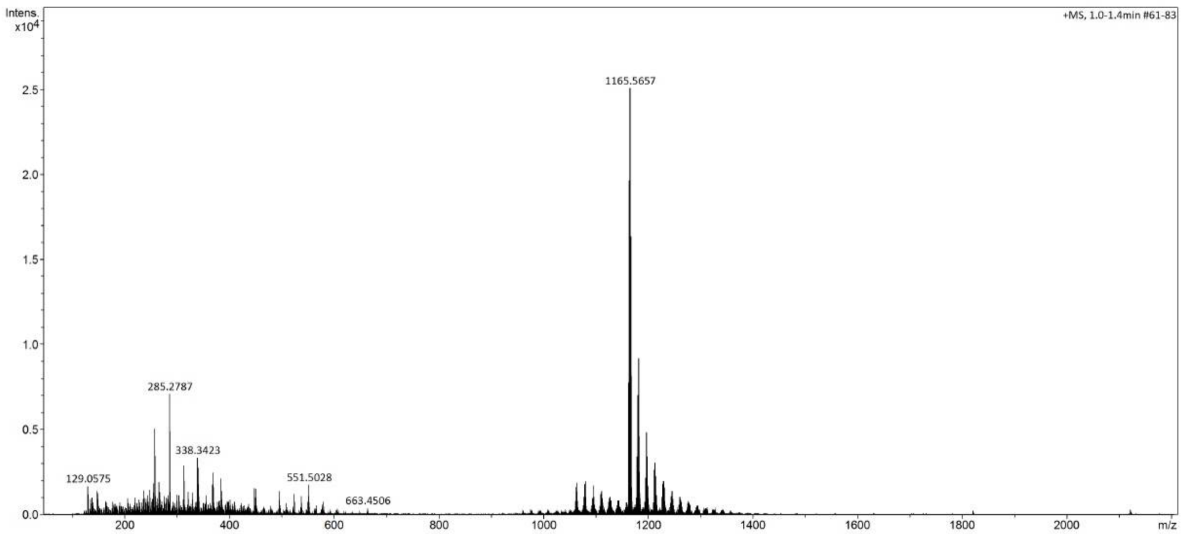

Figure **S8**. HRMS of **NOBNacene.**

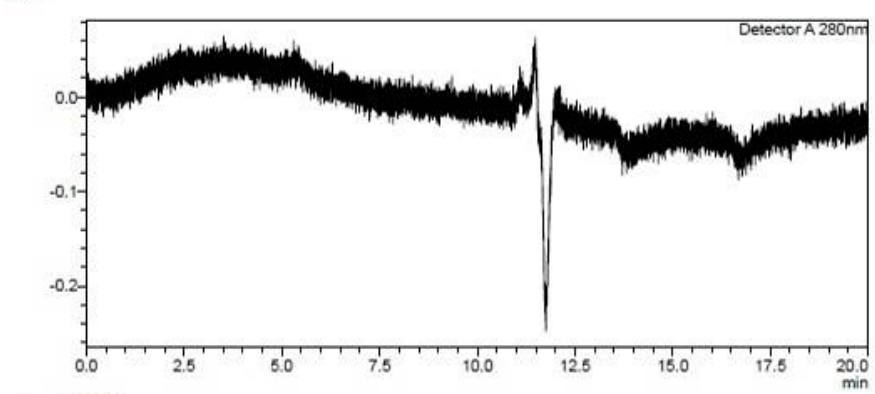

HPLC Trace Report28Jan2022

<Sample Information>

Sample Name	: blank		
Sample ID	:		
Method Filename	: 100% THF 10 mins 280nm.lcm		
Batch Filename	: 28012022.lcb		
Vial #	: 1-1	Sample Type	: Unknown
Injection Volume	: 5 uL		
Date Acquired	: 28/01/2022 14:18:28	Acquired by	: System Administrator
Date Processed	: 28/01/2022 14:38:29	Processed by	: System Administrator

<Chromatogram>

<Peak Table>

Detector A 280nm

Peak#	Ret. Time	Area	Height	Area%	Area/Height	Width at 5% Height
Total						

Figure **S9**. GPC trace of THF blank.

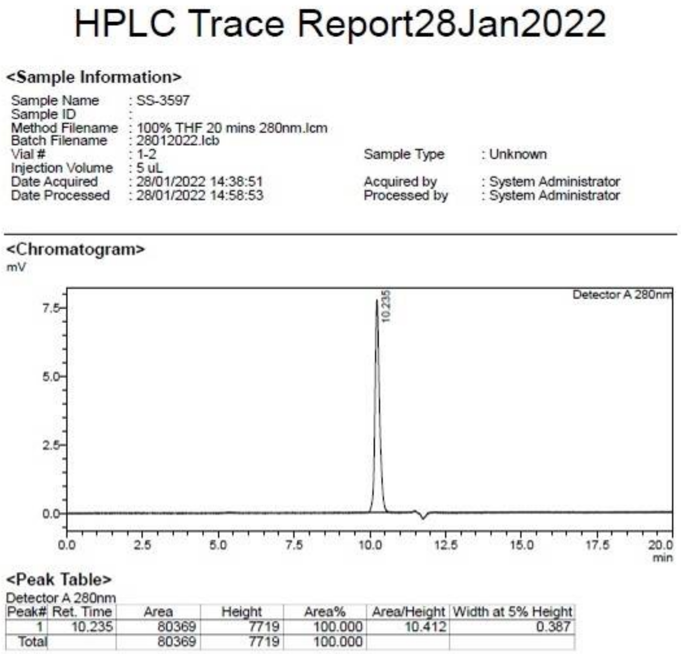

Figure **S10**. HPLC-GPC trace of **NOBNacene** after subtracting blank peak.

Table S1. Excited-state energies and nature in terms of one-electron contributions calculated at the SCS-CC2/cc-pVDZ level of theory of **NOBNacene** from vertical excitation from the PBE0/6-31G(d,p) ground-state-optimized geometry.

**Excited state**	**Energy / eV**	***f*[a]**	**Nature[b]**
T_1	3.40	N/A	H-1→L+2 65%, H-1→L+1 10%, H→L 4%, H-11→L+2 2%, H-17→L+2 2%
T_2	3.47	N/A	H→L 46%, H-2→L+1 13%, H-1→L+2 6%, H-3→L+1 6%, H-2→L+2 5%, H→L+3 4%, H-3→L+2 3%, H→L+2 2%, H-6→L+3 2%
S_1	3.52	0.32	H→L+2 47%, H→L+1 12%, H-1→L 10%, H-1→L+3 9%, H-6→L+2 7%, H-1→L+3 3%
S_2	3.57	0.00	H-1→L+2 43%, H→L 25%, H-1→L+1 8%, H-3→L+1 5%, H→L+1 3%, H-10→L+2 2%

[a]Oscillator strength, [b] H is HOMO and L is LUMO.

Table S2. Excited-state energies and nature in terms of one-electron contributions calculated at the SCS-CC2/cc-pVDZ level of theory of **NOBNacene** from the TDA-PBE0/6-31G(d,p) excited-state optimized geometry.

Excited state	Energy / eV	f^a	Nature[b]
T_1[c]	3.28	N/A	H→L 48%, H-2→L 16%, H→L+2 7%, H-2→L+1 4%, H→L+3 3%, H→L+1 2%
S_1	3.46	0.40	H→L+2 40%, H→L 15%, H-2→L 6%, H-1→L+3 5%, H-1→L+1 4%, H-1→L+2 4%, H→L+1 3%, H-7→L+2 3%, H-3→L 2%, H-7→L 2%, H-1→L+1 2%

[a]Oscillator strength, [b] H is HOMO and L is LUMO, [c]From the T_1 optimized geometry, [d]From the S_1 optimized geometry.

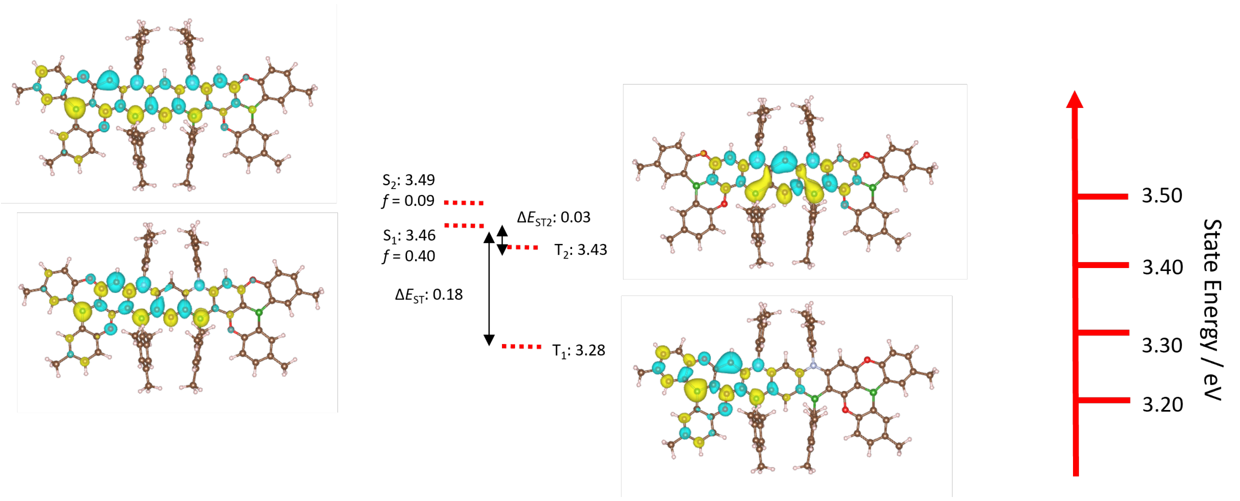

Figure **S11**. Energies and difference density plots of **NOBNacene** at the optimized S_1 and T_1 geometries calculated at SCS-CC2/cc-pVDZ level of theory (isovalue = 0.001).

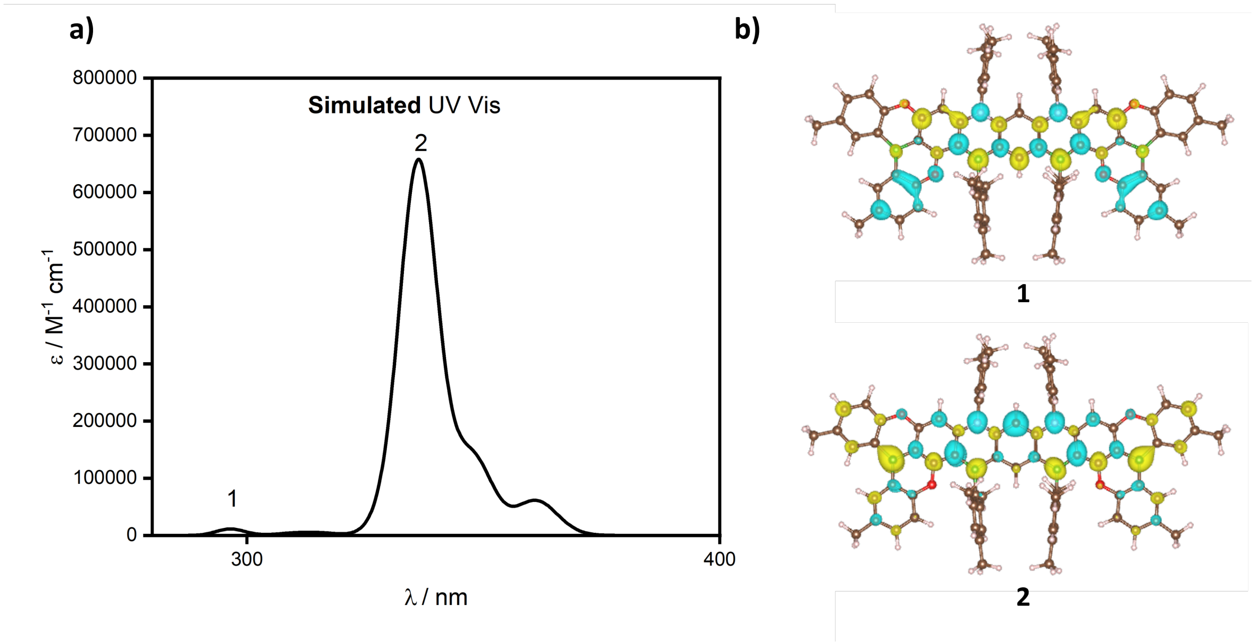

Figure **S12**. Simulated UV-Vis (**a**) and difference density plots (**b**) of important high energy transitions, calculated at TDA-PBE0/6-31G(d,p).

The simulation of the vibronically-resolved emission spectra has been carried out within the undistorted harmonic model, neglecting the Duschinsky rotation and assuming equal frequencies and normal modes for the ground- and excited-state. The intensity associated with each vibronic transition can be written as follow:

$$I_{i,0\rightarrow f,v_f} \propto \left|\vec{\mu}_{if}^{0}\right|^2 \prod_{k}^{TS} \frac{(S_k)^{n_k}}{n_k!} exp\,(-S_k)$$

in which n_k is the vibrational quantum number and S_k is the Huang-Rhys (HR) factor associated with the *k*-th vibrational normal mode, defining the projection of the geometry displacement between state i and f along the *k*-th totalsymmetric normal mode:

$$S_k = \frac{1}{2}\left\{\sqrt{\frac{\omega_k}{\hbar}}\left[X_i - X_f\right]\boldsymbol{M}^{\frac{1}{2}}\boldsymbol{L}_k(f)\right\}^2$$

in which ω_k is the vibrational frequency of the k-th normal mode, X_i and X_f are the cartesian coordinates of the equilibrium geometry of state state i and f, $\boldsymbol{M}$ is the diagonal matrix whose elements are the atomic masses, $\boldsymbol{L}_k(f)$ is the vector containing the normal coordinates written in terms of mass-weighted cartesian coordinate of the final state. The projection of the geometry displacement has been carried out on the ground-state vibrational normal modes computed at the PBE0/6-31G(d,p) level of theory. The HR factors have been calculated using MOMAP software.[12]

The resulting stick-spectra is a combination of each vibronic transition, which is then convoluted employing a Gaussian function. The broadening parameter (1200 cm^{-1}) has been chosen by fitting the simulated spectra to the experimental one.

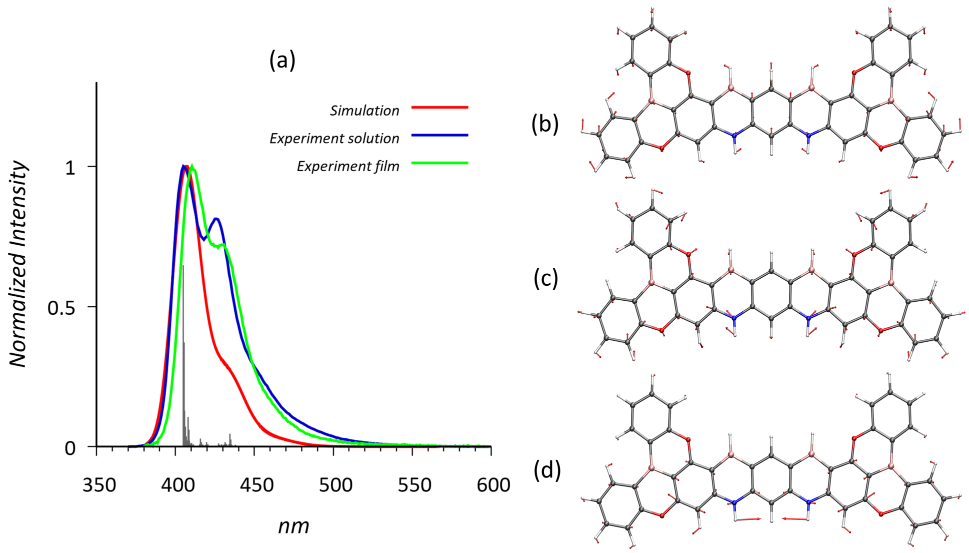

Figure **S13.** (a) simulated (red), THF (blue) and evaporated thin-film (green) emission spectra. (b) normal mode at 178 cm^{-1}, (c) 643 cm^{-1} and (d) 1674 cm^{-1}.

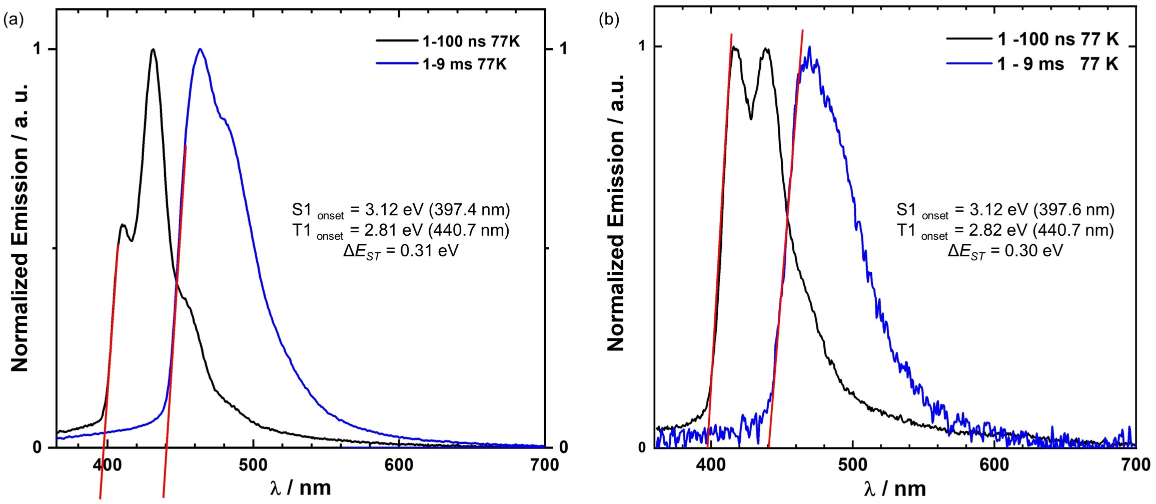

Figure **S14**. Prompt fluorescence and phosphorescence spectra at 77 K by using an iCCD. The integration time was 1-100 ns and 1-9 ms after the excitation laser pulse (λ_{exc} = 343 nm) for prompt fluorescence and phosphorescence spectra, respectively. a) measured in 10^{-6} M **NOBNacene** in 2Me-THF b) measured in vacuum-evaporated thin films on sapphire substrates (thickness: 60 nm) with 1.5 wt% doping of **NOB-Nacene** in TSPO1 host. The S_1 and T_1 exciton energies were determined from the onset of the spectrum.

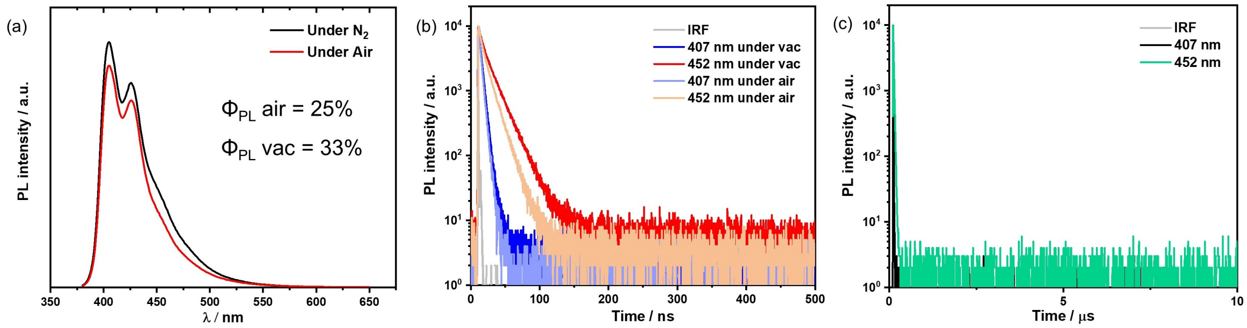

Figure **S15 a**) Steady-state emission spectra in deaerated and aerated dilute 2-THF solutions. **b**) and **c**) are decay of emission signal at RT under deaerated and aerated conditions using TCSPC and MCS for **b**) and **c**) respectively. IRF is instrumental response function λ_{exc} = 378 nm.

Ambient-pressure photoemission spectroscopy (APS) for HOMO energy level

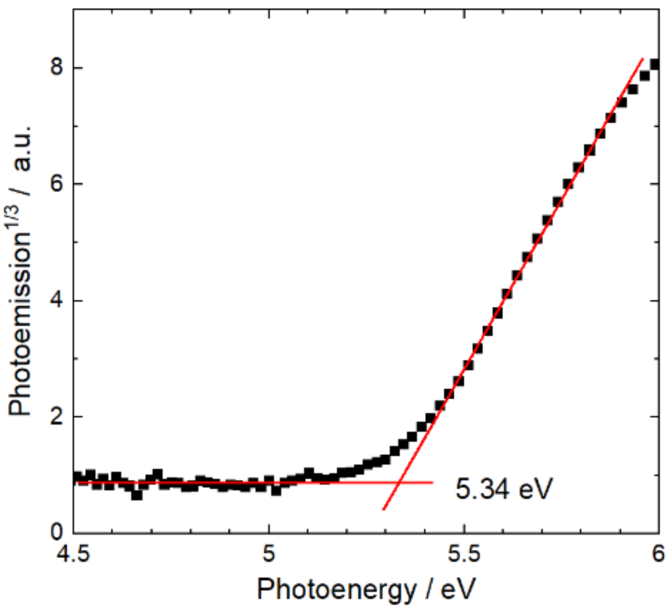

Figure **S16**. Ambient pressure photoemission spectroscopy (APS) for **NOBNacene** on an ITO substrate. The HOMO level was determined by extrapolating the linear part of the curve down to the baseline.

The HOMO level of **NOBNacene** was estimated by using ambient-pressure photoemission spectroscopy with a high energy resolution of 50 meV (APS04, KP Technology Ltd.). The sample was a 20 nm thick neat **NOBNacene** thin film that was vacuum-deposited on a conducting ITO substrate. The substrate was carefully grounded to avoid the detrimental effect of charge accumulation during the measurement. The excitation photon energy was scanned from 4.5 eV to 6.5 eV, during which the photoemission current (I_{ph}) was recorded. In Figure **S16**, the cubic root of I_{ph} is shown with respect to the excitation photon energy, which exhibits a linear relationship above the baseline, typical for semiconducting materials. From the extrapolation to the baseline, the HOMO energy level was estimated as 5.34 eV, which is lower than the HOMO values predicted from the ground state gas phase calculation (-4.99 eV). With the optical band gap determined from the intersection of the normalized absorption and emission spectra in solution (E_{opt} = 3.14 eV), the LUMO level was inferred to be -2.20 eV.

Table **S3**. Photoluminescence quantum efficiency, Φ_{PL} of **NOBNacene** doped in various hosts at the doping concentration of 1.5 wt%.

Emitter	Φ_{PL} in different Hosts[a] / %			
	TSPO1	DPEPO	PPT	mCP
**NOBNacene**	71	67	56	41

[a]measurements were performed by using an integration sphere under N_2 flow at room temperature. $\lambda_{exc.}$ = 290 nm.

Device Fabrication.

All functional materials were purchased from Lumtec and used as received. Devices were fabricated by thermal evaporation under a high vacuum with the base pressure less than 10^{-6} mbar (Angstrom evaporator, located inside a standard glove box with O_2, H_2O < 0.1 ppm). The film thickness was monitored during evaporation by using a QCM. Patterned ITO/glass substrates (ITO thickness = 90 nm, R_s < 30 ohms/sq) were used as anode, which were cleaned before film deposition by sonication in acetone, IPA for 15 min, and then treated by oxygen plasma for 3 min. The device size is determined by the shadow mask, which is 2 mm^2. After fabrication, all samples were encapsulated inside the glove box.

The current-voltage-luminance (*J-V-L*) characteristics were measured at room temperature using a Keithley 2400 source meter combined with a calibrated Si-photodiode connected to a multimeter (Keithley 2000) for the photovoltage reading. The external quantum efficiency (EQE) and luminance (*L*) are calculated assuming Lambertian emission distribution. The electroluminescence spectra were obtained by an Andor DV420-BU CCD spectrometer.

The host material was selected among four widely used high triplet energy hosts, i.e., mCP (*N,N'*-dicarba-zolyl-3,5-benzene, E_T = 2.9 eV),[13] PPT (2,8-bis(diphenyl-phosphoryl)-dibenzo[b,d]thiophene, E_T = 3.1 eV),[14] DPEPO (bis[2-(diphenylphosphino)phenyl]ether oxide, E_T = 3.0 eV),[15] and TSPO1 (diphenyl[4-(triphenylsilyl)phenyl]phosphine oxide, E_T = 3.36 eV).[16] The photoluminescence quantum yield, Φ_{PL} of vacuum evaporated **NOBNacene** doped thin films (thickness: 60 nm) at a doping concentration of 1.5 wt% was measured by using an integration sphere under N_2 flow at room temperature. Under the excitation

wavelength of 280 nm, Φ_{PL} was estimated to be 41%, 56%, 67%, and 71% for the host of mCP, PPT, DPEPO, and TSPO1, respectively.

The optimized device structure is oxide ITO/hexaazatriphenylenehexacarbonitrile (HAT-CN, 5 nm)/*N,N′*-di(1-naphthyl)-*N,N′*-diphenyl-(1,1′-biphenyl)-4,4′-diamine (NPB, 40 nm)/tris(4-carbazoyl-9-ylphenyl)amine (TCTA, 10 nm)/CzSi (10 nm)/ **NOBNacene** : TSPO1 or DPEPO (c_D = 1.5wt%, 3.0 wt%, and 6.0 wt%, 20 nm)/TSPO1 (10 nm)/ 1,3,5-tris(3-pyridyl-3-phenyl)benzene (TmPyPB, 20 nm)/LiF (0.8 nm)/Al (100 nm), where HAT-CH, NPB, TCTA, CzSi, TSPO1, TmPyPB, LiF are the hole injection, hole transport, electron blocking, exciton blocking, hole blocking, electron transport, and electron injection layers, respectively.

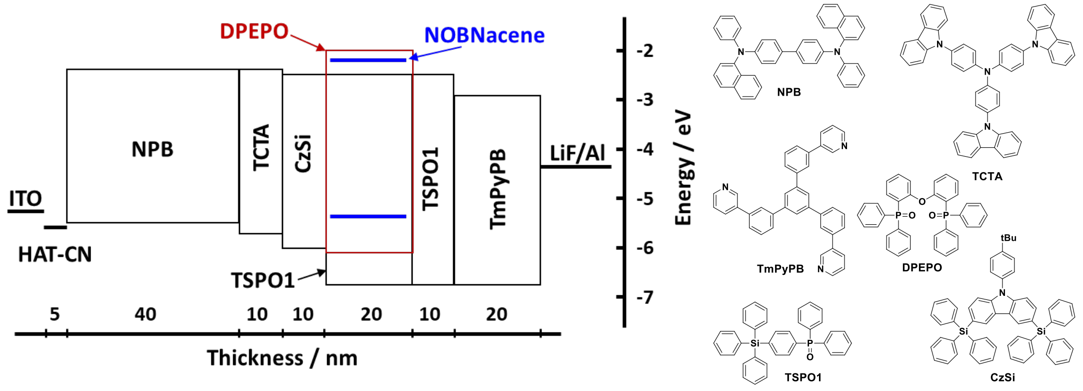

Figure **S17.** The optimized device structure with energy levels, thickness of each layer, and molecular structures indicated. The host was either DPEPO (in red) or TSPO1 (in black).

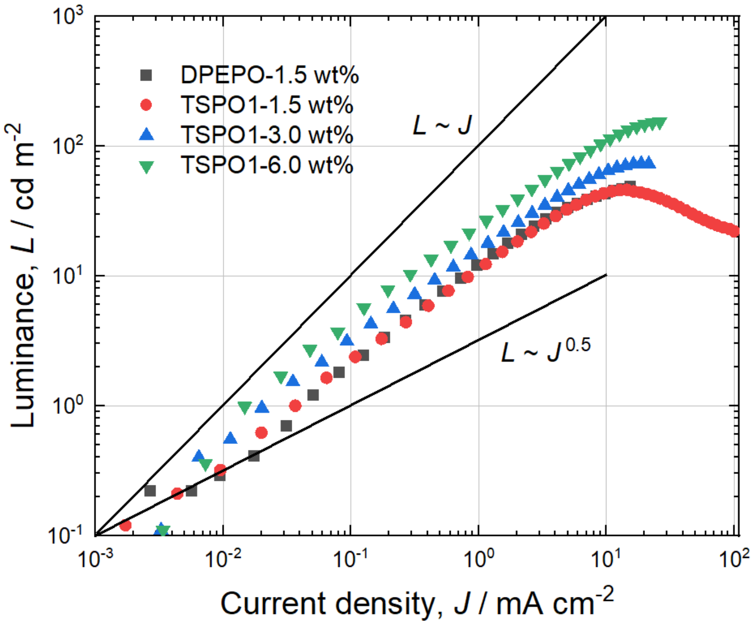

Figure **S18.** The relationship between luminance (*L*) and current density (*J*) of devices. For a clear comparison, the linear and quadratic dependence were indicated as well. At the same doping concentration of 1.5 wt%, the *J-L* curves of TSPO1 device and DPEPO device overlap with each other, indicating important role of **NOBNacene** on exciton generation and utilization. At low current density, all curves show a linear dependence, which deviated with increasing current density, implying possible exciton losses due to annihilation and quenching processes. The dependence rules out the possibility of TTA.

Table **S4**. EQE_{max} Data for TADF devices with $CIE_y < 0.1$ and $\lambda_{EL} < 435$ nm.

Emitter	CIE_y	λ_{EL}	EQE_{max}	Ref
**NOBNacene**	**0.055**	**409**	**8.5**	**This work**
	**0.068**	**412**	**11.2**	
	**0.103**	**412**	**10.2**	
BD1	0.05	424	8.9	[17]
BD2	0.06	430	9.5	
BD3	0.06	432	12	
BAn-(3,5)-CF3	0.083	435	5.02	[18]
PPINCN	0.08	431	3.38	[19]
PPINCN-Cz	0.08	430	9.89	
DSiTPI (nondoped)	0.06	406	5.3	[20]
CSiTPI (nondoped)	0.06	404	7.1	
DSiTPI (doped)	0.05	396	7.4	
CSiTPI (doped)	0.05	388	5.2	

DSiPPI	0.078	377	7.07	[21]
CNNPI	0.073	432	2.28	[22]
2FPPICz	0.045	425	4.3	[23]
NPBI-PPI-TPA	0.05	429	5.59	[24]
CzB-FMPIM	0.07	425	3.17	[25]
PI-NA	0.035	412	2.43	[26]
DPACTPI	0.068	428	2.31	[27]
DPACPhTPI	0.047	428	3.5	
DPACFPPI	0.057	424	3.03	
TPIBCz	0.046	432	5.46	[28]
TPIBNCz	0.048	428	5.99	
TPI	0.076	420	0.96	[29]
TPI-Bz	0.043	420	1.45	
TPI-2Na	0.063	428	1.98	
TPI-Ph	0.05	428	3.72	
PIMNA	0.034	412	2.43	[30]
m-BBTPI	0.06	428	3.63	[31]
BPA-BPI	0.05	428	1.37	[32]
PATPA	0.06	424	0.72	
BD-3a	0.046	422	2.99	[33]
BD-3b	0.04	422	2.97	
BD-3c	0.043	423	2.97	
TFPy2	0.03	414	3.8	[34]
TFPy3	0.02	406	4.8	
TFPy4	0.02	404	5.1	
BD-5	0.024	412	3.19	[35]
BD1	0.079	431	4.71	[36]
m-FLDID	0.027	411	5.2	[37]
tDIDCz	0.019	402	3.3	[38]
ICz-DPS	0.08	435	12.8	[39]
CZ-MPS	0.065	389	9.3	[40]
tBDCz-DPS	0.07	423	9.9	[41]
DCzBN1	0.05	418	2.5	[42]
DCzBN3	0.06	428	10.3	
CTPPI	0.04	396	7.9	[43]
CBPI	0.06	428	5.4	

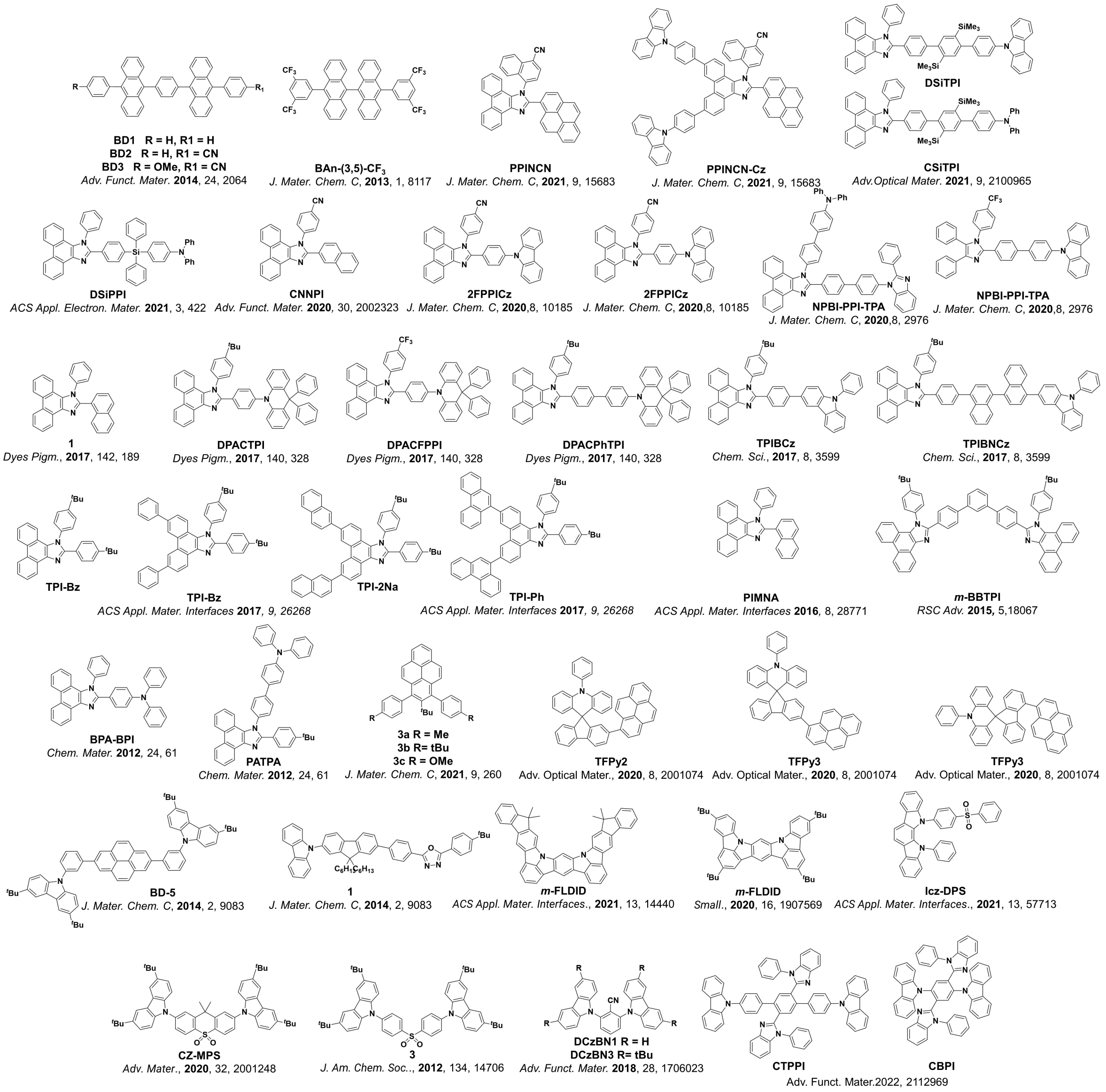

Figure **S19**. Chemical structures of the emitters given in the Table **S4**.

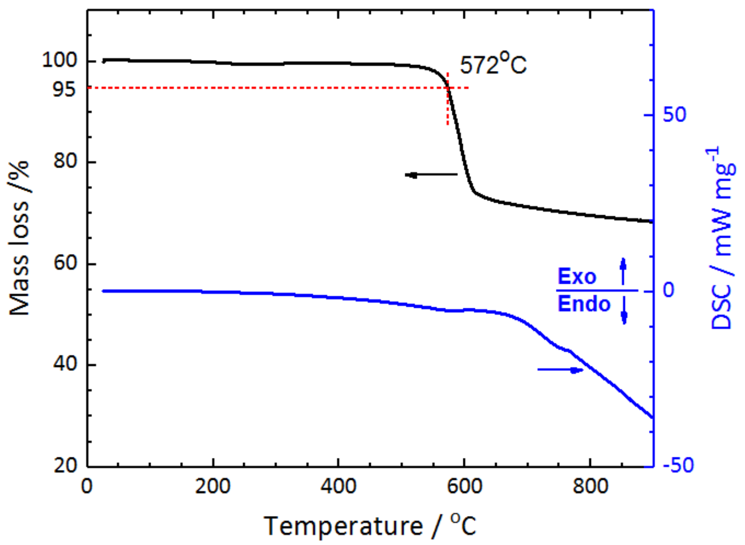

Figure **S20**. TGA/DSC results of **NOBNacene**. The mass loss of 5% of initial weight, T_d occurs at 572 °C. The TGA/DSC set-up is NETZSCH STA 449 C. The measurement was performed with continuous N_2 purge and at a temperature rising rate of 10 °C/min from room temperature to 900 °C.

References

[1] G. A. Crosby, J. N. Demas, *J. Phys. Chem.* **1971**, *75*, 991-1024.

[2] W. H. Melhuish, *J. Phys. Chem.* **1961**, *65*, 229-235.

[3] G. W. T. M. J. Frisch, H. B. Schlegel, G. E. Scuseria, M. A. Robb, J. R. Cheeseman, G. Scalmani, V. Barone, G. A. Petersson, H. Nakatsuji, X. Li, M. Caricato, A. Marenich, J. Bloino, B. G. Janesko, R. Gomperts, B. Mennucci, H. P. Hratchian, J. V. Ortiz, A. F. Izmaylov, J. L. Sonnenberg, D. Williams-Young, F. Ding, F. Lipparini, F. Egidi, J. Goings, B. Peng, A. Petrone, T. Henderson, D. Ranasinghe, V. G. Zakrzewski, J. Gao, N. Rega, G. Zheng, W. Liang, M. Hada, M. Ehara, K. Toyota, R. Fukuda, J. Hasegawa, M. Ishida, T. Nakajima, Y. Honda, O. Kitao, H. Nakai, T. Vreven, K. Throssell, J. A. Montgomery, Jr., J. E. Peralta, F. Ogliaro, M. Bearpark, J. J. Heyd, E. Brothers, K. N. Kudin, V. N. Staroverov, T. Keith, R. Kobayashi, J. Normand, K. Raghavachari, A. Rendell, J. C. Burant, S. S. Iyengar, J. Tomasi, M. Cossi, J. M. Millam, M. Klene, C. Adamo, R. Cammi, J. W. Ochterski, R. L. Martin, K. Morokuma, O. Farkas, J. B. Foresman, and D. J. Fox, Gaussian, Inc., Wallingford CT, 2016.

[4] C. Adamo, V. Barone, *J. Chem. Phys.* **1999**, *110*, 6158-6170.

[5] G. A. Petersson, M. A. Al-Laham, *J. Chem. Phys.* **1991**, *94*, 6081-6090.

[6] J. Thorn H. Dunning, *J. Chem. Phys.* **1989**, *90*, 1007-1023.

[7] C. Hättig, F. Weigend, *J. Chem. Phys.* **2000**, *113*.

[8] C. Hättig, K. Hald, *Phys. Chem. Chem. Phys.* **2002**, *4*, 2111-2118.

[9] a)T. Etienne, X. Assfeld, A. Monari, *J. Chem. Theory Comput.* **2014**, *10*, 3896-3905; b)T. Etienne, X. Assfeld, A. Monari, *J. Chem. Theory Comput.* **2014**, *10*, 3906-3914.

[10] R Dennington, T. Keith, J. Millam, KS, Semichem Inc.: Shawnee Mission, **2019**.

[11] K. Momma, F. Izumi, *J. Appl. Crystallogr.* **2011**, *44*, 1272-1276.

[12] Y. Niu, W. Li, Q. Peng, H. Geng, Y. Yi, L. Wang, G. Nan, D. Wang, Z. Shuai, *Mol. Phys.* **2018**, *116*, 1078-1090.

[13] Y. Kawamura, K. Goushi, J. Brooks, J. J. Brown, H. Sasabe, C. Adachi, *Appl. Phys. Lett.* **2005**, *86*, 071104.

[14] K. Goushi, C. Adachi, *Appl. Phys. Lett.* **2012**, *101*, 023306.

[15] J. Zhang, D. Ding, Y. Wei, H. Xu, *Chemical Science* **2016**, *7*, 2870-2882.

[16] S. O. Jeon, S. E. Jang, H. S. Son, J. Y. Lee, *Adv. Mater.* **2011**, *23*, 1436-1441.

[17] J.-Y. Hu, Y.-J. Pu, F. Satoh, S. Kawata, H. Katagiri, H. Sasabe, J. Kido, *Adv. Funct. Mater.* **2014**, *24*, 2064-2071.

[18] Y. Yu, Z. Wu, Z. Li, B. Jiao, L. Li, L. Ma, D. Wang, G. Zhou, X. Hou, *J. Mater. Chem. C* **2013**, *1*, 8117-8127.

[19] J. Jayabharathi, J. Anudeebhana, V. Thanikachalam, S. Sivaraj, *J. Mater. Chem. C* **2021**, *9*, 15683-15697.

[20] Y. Zheng, X. Zhu, Z. Ni, X. Wang, Z. Zhong, X. J. Feng, Z. Zhao, H. Lu, *Adv. Opt. Mater.* **2021**, *9*, 2100965.

[21] Y. Zheng, Z. Wang, X. Wang, J. Li, X. J. Feng, G. He, Z. Zhao, H. Lu, *ACS Appl. Electron. Mater.* **2021**, *3*, 422-429.

[22] H. Zhang, B. Zhang, Y. Zhang, Z. Xu, H. Wu, P.-A. Yin, Z. Wang, Z. Zhao, D. Ma, B. Z. Tang, *Adv. Funct. Mater.* **2020**, *30*, 2002323.

[23] J. Xin, Z. Li, Y. Liu, D. Liu, F. Zhu, Y. Wang, D. Yan, *J. Mater. Chem. C* **2020**, *8*, 10185-10190.

[24] J.-J. Zhu, Y. Chen, Y.-H. Xiao, X. Lian, G.-X. Yang, S.-S. Tang, D. Ma, Y. Wang, Q.-X. Tong, *J. Mater. Chem. C* **2020**, *8*, 2975-2984.

[25] Y. Jia, S. Wu, Y. Zhang, S. Fan, X. Zhao, H. Liu, X. Dong, S. Wang, X. Li, *Org. Electron.* **2019**, *69*, 289-296.

[26] T. Shan, Z. Gao, X. Tang, X. He, Y. Gao, J. Li, X. Sun, Y. Liu, H. Liu, B. Yang, P. Lu, Y. Ma, *Dyes Pigm.* **2017**, *142*, 189-197.

[27] Z. Huang, B. Wang, Q. Zhang, S. Xiang, X. Lv, L. Ma, B. Yang, Y. Gao, L. Wang, *Dyes Pigm.* **2017**, *140*, 328-336.

[28] W.-C. Chen, Y. Yuan, S.-F. Ni, Q.-X. Tong, F.-L. Wong, C.-S. Lee, *Chem. Sci.* **2017**, *8*, 3599-3608.

[29] W.-C. Chen, Y. Yuan, Y. Xiong, A. L. Rogach, Q.-X. Tong, C.-S. Lee, *ACS Appl. Mater. Interfaces* **2017**, *9*, 26268-26278.

[30] T. Shan, Y. Liu, X. Tang, Q. Bai, Y. Gao, Z. Gao, J. Li, J. Deng, B. Yang, P. Lu, Y. Ma, *ACS Appl. Mater. Interfaces* **2016**, *8*, 28771-28779.

[31] W.-C. Chen, G.-F. Wu, Y. Yuan, H.-X. Wei, F.-L. Wong, Q.-X. Tong, C.-S. Lee, *RSC Adv.* **2015**, *5*, 18067-18074.

[32] Y. Zhang, S.-L. Lai, Q.-X. Tong, M.-F. Lo, T.-W. Ng, M.-Y. Chan, Z.-C. Wen, J. He, K.-S. Jeff, X.-L. Tang, W.-M. Liu, C.-C. Ko, P.-F. Wang, C.-S. Lee, *Chem. Mater.* **2012**, *24*, 61-70.

[33] H. Ran, Z. Zhao, X. Duan, F. Xie, R. Han, H. Sun, J.-Y. Hu, *J. Mater. Chem. C* **2021**, *9*, 260-269.

[34] S.-N. Zou, X. Chen, S.-Y. Yang, S. Kumar, Y.-K. Qu, Y.-J. Yu, M.-K. Fung, Z.-Q. Jiang, L.-S. Liao, *Adv. Opt. Mater.* **2020**, *8*, 2001074.

[35] D. Chercka, S.-J. Yoo, M. Baumgarten, J.-J. Kim, K. Müllen, *J. Mater. Chem. C* **2014**, *2*, 9083-9086.

[36] K. E. Linton, A. L. Fisher, C. Pearson, M. A. Fox, L.-O. Pålsson, M. R. Bryce, M. C. Petty, *J. Mater. Chem.* **2012**, *22*, 11816-11825.

[37] V. V. Patil, J. Lim, J. Y. Lee, *ACS Appl. Mater. Interfaces* **2021**, *13*, 14440-14446.

[38] H. L. Lee, W. J. Chung, J. Y. Lee, *Small* **2020**, *16*, 1907569.

[39] J. Wang, J. Zhang, C. Jiang, C. Yao, X. Xi, *ACS Appl. Mater. Interfaces* **2021**, *13*, 57713-57724.

[40] Y. Luo, S. Li, Y. Zhao, C. Li, Z. Pang, Y. Huang, M. Yang, L. Zhou, X. Zheng, X. Pu, Z. Lu, *Adv. Mater.* **2020**, *32*, 2001248.

[41] Q. Zhang, J. Li, K. Shizu, S. Huang, S. Hirata, H. Miyazaki, C. Adachi, *J. Am. Chem. Soc.* **2012**, *134*, 14706-14709.

[42] C.-Y. Chan, L.-S. Cui, J. U. Kim, H. Nakanotani, C. Adachi, *Adv. Funct. Mater.* **2018**, *28*, 1706023.
[43] Z. Zhong, X. Zhu, X. Wang, Y. Zheng, S. Geng, Z. Zhou, X. J. Feng, Z. Zhao, H. Lu, *Adv. Funct. Mater.*, *n/a*, 2112969.
